# Metal-Coordinated His-Tag Functionalization of Polymeric Nanogels for Therapeutic Applications

**DOI:** 10.1021/acsanm.5c05531

**Published:** 2026-03-12

**Authors:** Camillo Colli, Andrea De Filippis, Gabriele Galbo, Martina Kunkl, Julia Ivanova, Susan Fornoni, Joachim P. Spatz, Laura Rosanò, Davide Moscatelli, Loretta Tuosto, Emanuele Mauri

**Affiliations:** a Department of Chemistry, Materials and Chemical Engineering “Giulio Natta”, 18981 Politecnico di Milano, piazza Leonardo Da Vinci 32, Milan 20133, Italy; b Department of Biology and Biotechnologies Charles Darwin, Istituto Pasteur Italia-Fondazione Cenci Bolognetti, 9311 Sapienza University of Rome, Rome 00185, Italy; c Department of Cellular Biophysics, 28296 Max Planck Institute for Medical Research, Bildungscampus Heilbronn, Heilbronn 74076, Germany; d Institute for Molecular Systems Engineering and Advanced Materials, Heidelberg University, Heidelberg 69120, Germany; e Max Planck School Matter to Life, Bildungscampus Heilbronn, Heilbronn 74076, Germany; f Institute of Molecular Biology and Pathology, 196021 National Research Council, Via degli Apuli 4, Rome 00185, Italy

**Keywords:** nanogels, His-tag, cobalt, poly(allylamine), drug delivery, ovarian cancer

## Abstract

Nanogels (NGs) are versatile polymeric nanocarriers with high drug loading capacity and colloidal stability, making them pivotal candidates for targeted biomedical applications. Surface functionalization with biomolecules is essential for selective targeting therapies; however, conventional covalent conjugation can compromise the activity of sensitive ligands (e.g., proteins and antibodies). The His-tag strategy offers a reversible, oriented binding through coordination with transition metal ions, widely used in protein purification, but scarcely explored for polymeric NG decoration. Here, we developed polyallylamine-based NGs via an emulsion–evaporation process. The NG outer layer was functionalized with lysine-conjugated nitrilotriacetic acid to chelate Ni^2+^ or Co^3+^ ions, enabling the His-tag strategy. To validate this approach, a His-Rhodamine compound was used as a representative His-tag structure, mimicking potential bioactive ligands or motifs. Our results demonstrate that Co^3+^ provides superior His-tag grafting density compared to Ni^2+^, preserving the biocompatibility of the nanoscaffold. The cobalt-complexed NGs were also tested as cisplatin delivery systems, showing enhanced therapeutic performance compared with the administration of the free drug in ovarian cancer cells. Overall, the cobalt-mediated His-tag conjugation proved to be an efficient approach for the noncovalent surface decoration of polymeric NGs, defining a reliable alternative for the functionalization of this type of nanoscaffolds. Owing to the presence of His-tag functionalities in several biomolecules, the proposed strategy can be readily extended to a wide range of His-tag-based species, thus providing a flexible platform for the design of advanced, targeted NGs with improved selectivity and therapeutic potential.

## Graphical abstract

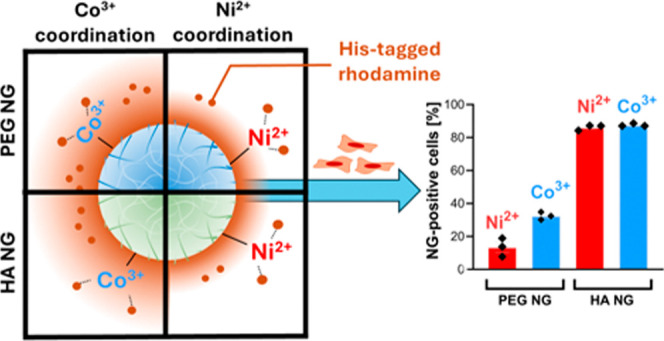

## 1 Introduction

In the last decades, nanomaterials have gained significant interest in the biomedical field due to their ability to cross biological barriers and accumulate in tumor tissues through their passive targeting effect (EPR effect), offering the potential to overcome limitations associated with conventional therapies.^1–3^ In particular, different types of polymer-based nanomaterials are extensively studied, such as micelles, dendrimers, liposomes, and nanoparticles,^4,5^ using both natural and synthetic polymers. The smart combination of these macromolecules may lead to “hybrid” nanostructures with superior properties compared to the corresponding individual components. Indeed, natural polymers (e.g., hyaluronic acid, pullulan, and chitosan) enhance the biocompatibility of the final system, able to structurally resemble biological architectures or stimulate cellular responses,^6,7^ while synthetic polymers enable control over physicochemical and mechanical properties, degradation rates, and composition, features that are difficult to tune using solely natural components. In this context, nanogels (NGs) are widely recognized as promising hybrid nanoscaffolds for different biomedical applications, ranging from tumor-related diseases (e.g., ovarian, lung, breast, pancreas, and colon) to theranostics.^8–11^

NGs are nanoscopic, cross-linked polymer networks, characterized by high stability in physiological conditions, good cell permeation capability, high surface-to-volume ratio, and swelling behavior. Thanks to their high drug loading ability, they are validated as drug delivery systems, enabling the encapsulation of both hydrophilic and hydrophobic compounds and controlling the drug release kinetics with a consequent reduction in systemic toxicity.^12,13^ For these reasons, they represent a potential nanoplatform for an advanced therapeutic strategy.

Furthermore, due to their mechanical properties and their capability of mimicking biofeatures, they are able to withstand hemodynamic shear forces and maintain stability in the presence of serum proteins within the circulatory system.^14^

However, the efficient performance of NGs is strictly correlated with their selective interaction with target sites in the biological microenvironment. To address this aspect, NGs are surface decorated with specific motifs (e.g., biomolecules, inhibitors, proteins, and modified PEGylation) mainly exploiting chemical-based conjugation methods, with the formation of new chemical bonds between the polymeric nanoscaffolds and the decoration. This grafting strategy is viable when the formation of a new linkage does not induce functional or structural alterations in the compound’s intrinsic activity. This has been demonstrated in the conjugation of polymers, some proteins/amino acids, or specific chemical compounds.^15,16^ However, in the case of recombinant proteins, tumor-targeting peptides, and antibodies, a permanent covalent bond may affect their biological or therapeutic functionality. In these cases, one leading strategy for assessing the nanoparticle coating is the use of histidine-tag (His-tag) portions.

His-tag consists of short sequences of histidine residues (usually six) that are engineered and fused to the N- or C- terminus of a protein or peptide structure. It exploits the affinity of the imidazole group to some transition metal ions (e.g., Ni^2+^, Zn^2+^, and Co^3+^)^17–19^ to generate an oriented and reversible conjugation. In particular, this approach has been validated for protein purification, immobilization and labeling,^20,21^ whereas its implementation in surface decoration of polymer-based nanoparticles is underexplored. Indeed, to date, most reported studies involve the use of inorganic nanoparticles, such as DOGS-Nickel systems, magnetic, gold nanoparticles,^22–24^ and silica nanoparticles coated with a polymeric layer to support His-tag engineering.^25,26^ Additionally, protein nanoparticles have been investigated exploiting the divalent metal cation coordination of polyhistidine-tagged building block proteins.^27^ On the other hand, Zhou et al.^28^ synthesized a hybrid nanoparticle for the intracellular delivery of proteins, by exploiting the His-tag conjugation. The nanoparticle was composed of methoxy-poly­(ethylene glycol)-*block*-poly­(l-phosphotyrosine) (mPEG-*b*-PpY)-templated calcium phosphate (CaP), and they performed protein coordination through interactions between Zn^2+^ and the histidine moieties of the protein. The results showed the capability of the nanosystems to correctly deliver proteins to the target cells. Tanner et al.^29^ developed polymer vesicles composed of a diblock copolymer (polybutadiene-*block*-polyetylenoxide) with nitriloacetic acid moieties for His-tagging proteins on their surface, exploiting the use of Cu^2+^ and Ni^2+^ metal ions for complexation.

Except for these examples, the His-tag potential in the context of polymeric NGs has not yet been investigated. The possibility of conjugating His-tagged molecules onto the NG surface could open new avenues for the development of innovative targeted therapies, combining the advantages of this class of nanocarriers with the selective interactions toward cell receptors and the enhanced intracellular drug delivery, thereby reducing systemic exposure. In particular, in this context, the unique features of NGs play a pivotal role. As soft nanosystems, NGs can efficiently interact with cell membranes, promoting adhesion and facilitating cellular internalization. Their intrinsic swelling behavior in aqueous environments, together with a high surface-to-volume ratio, further enhances their capacity to host and release therapeutic agents. Additionally, the wide choice of polymeric building blocks represents a key asset to finely tune the formation and physicochemical properties of the network, allowing their design to be adapted according to the specific application requirements and the temporal control over drug release.^30^

In this work, we proposed novel formulations of polyallylamine-based NGs and validated the procedure for their surface decoration with His-tag compounds, using two different metal ions: Ni^2+^ and Co^3+^.

In particular, we synthesized three types of NGs through the chemical cross-linking between poly­(allylamine hydrochloride) and carboxymethyl pullulan, hyaluronic acid, and poly­(ethylene glycol), respectively. NG synthesis was performed by exploiting a mixed emulsion-evaporation technique^9,31^ and characterized in terms of size, composition, and colloidal stability. Successively, NGs were decorated with l-lysine-conjugated nitriloacetic acid (lys-NTA) as a chelating agent for the metal ions, and a His-tag rhodamine (His-Rhod) was complexed onto the NGs exploiting the metal coordination mechanism. His-Rhod was chosen as a representative His-tag-modified compound for NG surface decoration.

In particular, we observed the high potential of Co^3+^ in enabling a higher grafting density of His-Rhod and enhancing the biocompatibility of the final nanocarriers, compared to the conventional use of Ni^2+^. These results confirmed the favorable ligand exchange properties of cobalt and its capability to form stable yet reversible complexes. Furthermore, we validated the cobalt-complexed NGs as an efficient cisplatin delivery system in ovarian cancer cells, demonstrating improved therapeutic outcomes compared to the administration of the free drug.

The proposed synthetic route provides a guideline for the use of NGs as nanoplatforms for His-tag binding, showing unprecedented surface attachment efficiency due to the employment of cobalt as an alternative to traditional transition metals. This strategy could be extended to the coordination of other His-tagged ligands, paving the way for novel targeted therapy formulations.

## 2 Materials and Methods

### 2.1 Materials

Hyaluronic acid sodium salt (HA, extra low MW = 8000–15,000) and pullulan (PUL, catalog no. YP07957) were purchased from Biosynth GmbH (Staad, Switzerland). Poly­(allylamine hydrochloride) (PAH, average MW = 17,500), poly­(ethylene glycol) 4000 (PEG-4k, MW = 4000), poly­(ethylene glycol) 8000 (PEG-8k, MW = 8000), 1,1′-carbonyldiimidazole (CDI, ≥97.0, MW = 162.15), dichloromethane (DCM, ≥99.8%, MW = 84.93), acetonitrile (ACN, ≥99.9%, MW = 41.05), *N*-hydroxysuccinimide (NHS, 98%, MW = 115.09), succinic anhydride (SA, ≥99%, MW = 100.07), 4-dimethylaminopyridine (DMAP, ≥99%, MW = 122.17), triethylamine (TEA, ≥99%, MW = 101.19), chloroform (≥99.5%, MW = 119.38), sodium chloride (NaCl, ≥99.0%, MW = 58.44), phosphate-buffered saline (PBS, tablets), propargyl bromide solution (80% w/w in toluene, MW = 118.96), copper­(II) sulfate (CuSO_4_, ≥99%, MW = 159.61), (+)-l-sodium ascorbate (NaAsc, ≥99.0%, MW = 198.11), methanol (≥99.8%, MW = 32.04), nickel chloride (NiCl_2_, 98%, MW = 129.60) Nα,Nα-bis­(carboxymethyl)-l-lysine hydrate (lys-NTA, ≥97.0%, MW = 262.26), TRIS buffer (TRIS, powder), l-histidine (His, 98.5–101.0%, MW = 155.15), rhodamine B base (Rhod, 97%, MW = 442.55), ethylenediamine (EDA, ≥99%, MW = 60.10), cobalt­(II) nitrate hexahydrate (≥98%, MW = 291.03), hydrogen peroxide solution (H_2_O_2_, 30% w/w, MW = 34.01), sodium bicarbonate (≥99.7%, MW = 84.01), ethanol (EtOH, ≥99.9%, MW = 46.07), diethyl ether (≥99.9%, MW = 74.12), 2-propanol (IPA, 99.9%, MW = 60.10), sodium hydroxide (NaOH, pellets, ≥98%, MW = 40.00), sodium chloroacetate (NaCA, ≥98.0%, MW = 116.48), thiazolyl blue tetrazolium bromide (MTT, 98%, MW = 414.32), dimethyl sulfoxide (DMSO, ≥99.9%, MW = 78.13), and ethylenediaminetetraacetic acid (EDTA, ≥99%, MW = 292.24) were purchased from Merck KGaA (Darmstadt, Germany). Cyanine5 azide (Cy5-azide, MW = 601.22) was purchased from Lumiprobe GmbH (Hannover, Germany). *N*-(3-(Dimethylamino)­propyl)-*N*′-ethylcarbodiimide hydrochloride (EDC, 99.0%, MW = 191.7), deuterium oxide + 0.002% TMSP (D_2_O, 99.9%, MW = 20.03), and chloroform D + 0.03% TMS­(CDCl_3_, ≥99.96%, MW = 120.38) were purchased from Fluorochem Ltd. (Hadfield, United Kingdom). Cisplatin (MW = 300.05) was purchased from D.B.A. Italia s.r.l. (Segrate, Italy). Spectrum Spectra/Por RC dialysis membranes were purchased from Thermo Fisher Scientific (Waltham, Massachusetts, USA). The Milli-Q Direct system (Merck KGaA, Darmstadt, Germany) was used to produce ultrapure water (DDIW) for the preparation of aqueous solutions. Solvents were of analytical grade purity, and reagents were used as received.

### 2.2 PAH-Cy5 Synthesis

PAH was functionalized with Cy5 and used in NG synthesis to ensure nanoscaffold traceability in flow cytometry experiments. Slightly modifying the procedure of our previous work,^31^ the first step consisted in the PAH functionalization with alkyne groups: PAH (250 mg, 0.014 mmol) was dissolved in a mixture of methanol and water (6:4, 10 mL), and propargyl bromide solution (16 μL, 0.14 mmol) was added dropwise at 0 °C and left under stirring for 24 h in the dark at room temperature (RT). The resulting solution was dialyzed against DDIW (MWCO = 3.5 kDa) for 2 days, and the product was obtained after freeze-drying. Then, PAH-alkyne (175 mg, 0.01 mmol) was dissolved in DDIW (7 mL) and Cy5-azide (200 μL, 2 mg/mL in DMSO) was added dropwise to the solution. CuSO_4_ (1.6 mg, 0.01 mmol) and NaAsc (2 mg, 0.01 mmol) were added, and the reaction was left under stirring for 36 h in the dark at 50 °C. Finally, the solution was dialyzed against DDIW (MWCO = 3.5 kDa) for 2 days and freeze-dried.

### 2.3 Pullulan Carboxymethylation

Pullulan carboxymethylation was carried out via etherification of its hydroxyl groups following a heterogeneous, multistep slurry process,^32,33^ under specific conditions. The synthesis was preceded by a swelling step in IPA at 20 °C for 1 h (10 g_IPA_/g_PUL_), to enhance the reactivity of the polymer. The swollen material was then basified by the dropwise addition of aqueous NaOH (30% w/w) into the reactor followed by stirring at 20 °C for 90 min to activate the −OH groups of the polysaccharide. A molar ratio of NaOH:PUL maltotriose units of 4.2:1 was used. The activated polymer was then etherified by gradually adding NaCA to the mixture (molar ratio NaCA:PUL maltotriose units 4.2:1) and leaving the reactive system under stirring at 70 °C for 90 min. The end-product composition contained a mixture of sodium salts in a basic IPA solution: precipitated sodium carboxymethyl pullulan (NaCMP), NaCl (byproduct), sodium glycolate (SG, byproduct), and potential residual NaOH and unreacted NaCA. To collect the functionalized NaCMP, the mixture was washed in a hot EtOH–water solution (80:20 v/v) for 10–15 min and then filtered. This procedure was repeated three times to ensure complete removal of the byproducts (soluble in the aqueous solution). Finally, the purified NaCMP was dried in a convective oven for 12 h at 60 °C to eliminate residual EtOH, IPA, and water. Characterization was performed via FTIR and ^1^H NMR in D_2_O.

### 2.4 Im-PEG-Im Synthesis

Im-PEG-Im was synthesized following the procedure reported in our previous works.^34^ Briefly: PEG-8k (2 g, 0.25 mmol) was dissolved in acetonitrile (20 mL), and CDI (405 mg, 2.5 mmol) was then added to the mixture. The reaction mixture was stirred at 40 °C for 17 h. Subsequently, the organic solvent was removed under reduced pressure using a rotary evaporator, and DDIW (5 mL) was added. The aqueous solution was dialyzed (MWCO = 3.5 kDa) against the DDIW for 48 h. The product was finally freeze-dried, obtaining a white powder. Characterization was performed via FTIR and ^1^H NMR in CDCl_3_.

### 2.5 COOH-PEG-COOH Synthesis

PEG-diacid was synthesized using PEG-4k and PEG-8k, adapting the reaction from our previous work.^9^ Referring to PEG-8k, the polymer (5.48 g, 0.7 mmol), SA (0.28 g, 2.8 mmol), and DMAP (36.7 mg, 0.3 mmol) were dissolved in DCM (20 mL). The mixture was left stirring for 24 h at RT. DCM was then evaporated under reduced pressure, and diethyl ether (20 mL) was added to precipitate the product. Finally, the functionalized PEG was collected as a dried powder after vacuum filtration. Characterization was performed via ^1^H NMR in CDCl_3._

### 2.6 Histidine-Rhodamine Synthesis

A histidine-functionalized Rhod derivative was synthesized as a model His-containing compound to validate His-tag-driven NG decoration and enable detection via applied characterization methods. The synthesis followed a two-step approach. The first one involved the activation of Rhod with the amine group.^35^ Briefly, Rhod (1.2 g, 2.5 mmol) was dissolved in EtOH (30 mL) and EDA (220 mL, 3.25 mmol) was added dropwise at RT, under stirring. Then, the mixture was heated to reflux for 72 h until the solution color changed to dark orange. The solvent was evaporated under reduced pressure, and the product (labeled as Rhod-NH_2_) was collected as solid, following precipitation in diethyl ether and filtration. The second step regards the conjugation of the histidine moiety. His (44 mg, 0.2 mmol), EDC (80 mg, 0.4 mmol), and NHS (36 mg, 0.31 mmol) were dissolved in acid water (pH = 4, 6 mL) and left under stirring for 90 min. Then, an acid solution (pH = 4, 4 mL) of Rhod-NH_2_ (100 mg, 0.2 mmol) was added dropwise to the His solution and left under vigorous stirring overnight at RT. The resulting system was washed with diethyl ether (3 × 6 mL) to remove unreacted Rhod, and residual solvent was removed under reduced pressure; the product (His-Rhod) was obtained after freeze-drying. Characterization was performed via^1^H NMR, FTIR, and HPLC.

### 2.7 Triscarbonatocobalt­(III) Salt Synthesis

Na_3_[Co^III^(CO_3_)_3_]·3 H_2_O salt (Co^3+^) was synthesized following the procedure patented by Benk and collaborators (WO2020254539)^36^ and described by Bauer and Drinkard,^37^ for the formation of the [Co^III^(NTA)­(His-Rhod)] complex. Cobalt­(II) nitrate hexahydrate (29.1 g, 0.1 mol) was dissolved into DDIW (50 mL), and H_2_O_2_ (10 mL) was added dropwise under continuous stirring to an ice-cooled sodium bicarbonate slurry (42 g, 0.5 mol) in DDIW (50 mL). The mixture was left stirring in ice bath for 1 h.

Subsequently the dark-green obtained product was filtered; washed with cold water (3×), EtOH (1×), and diethyl ether (1×); and vacuum-dried overnight. The salt was stored at −20 °C in N_2_ atmosphere. To produce the solution, the salt was dissolved in 1 M NaHCO_3_ by sonication. The solution was then filtered (0.22 μm) and stored at 4 °C.

### 2.8 Nanogel Synthesis

#### 2.8.1 PAH-HA and PAH-CMP Formulation

NGs composed of PAH and HA were synthesized according to the mixed emulsion/evaporation technique (MEET) reported in our previous work^31^ with a molar ratio PAH:HA equal to 1:0.75. Briefly, HA (20 mg, 0.053 mmol), EDC (50.71 mg, 0.265 mmol), and NHS (12.18 mg, 0.106 mmol) were dissolved in physiological solution (NaCl 0.9% w/v in DDIW, 4 mL) at a molar ratio of COOH_HA_:EDC:NHS of 1:5:2 at RT and kept under stirring for 90 min. DCM (7.5 mL) was added dropwise to the HA solution and sonicated for 30 min, generating the first W/O emulsion. Then, PAH-Cy5 (2.3 mg, 0.04 mmol) was separately dissolved in physiological solution (2 mL) and added dropwise to the emulsion. The resulting mixed emulsion, with two disperse aqueous phases, was sonicated for 15 min and subsequently stirred vigorously at RT overnight, allowing the organic solvent to evaporate and the coalescence of the aqueous phases. Finally, the aqueous solution was dialyzed against DDIW (MWCO = 12–14 kDa) for 2 days with daily water exchange and freeze-dried, and NGs were collected as a sponge-like solid.

The same procedure was applied for the synthesis of PAH-CMP NGs, where NaCMP (DS = 0.57, 20 mg, 0.032 mmol) was used instead of HA, and activated with EDC (54 mg, 0.281 mmol) and NHS (12.97 mg, 0.113 mmol). The molar ratio between the condensing-coupling agents and the polymer was set with reference to the CMP carboxyl groups. In NG synthesis, the molar ratio between the reactive groups of the polymers (COOH_NaCMP_:NH_2 PAH_) was set at 1:0.75 and PAH (2.4 mg, 0.042 mmol) was treated as previously described.

#### 2.8.2 PAH-PEG Formulation

Alternatively, the synthesis of PAH-PEG NGs was performed via the emulsion/evaporation method, exploiting the solubility of PEG in organic solvent.^38^ Briefly, Im-PEG-Im (50 mg, 6.25 μmol) was dissolved in DCM (5 mL), while PAH-Cy5 (21.88 mg, 1.25 μmol) was dissolved in physiological solution (3 mL). PEG organic solution was added dropwise to the PAH-Cy5 aqueous phase and sonicated for 30 min, generating a W/O emulsion, which was left under vigorous stirring overnight, at RT, until complete evaporation of the organic phase. The remaining solution was dialyzed against DDIW (MWCO = 12–14 kDa) for 2 days with daily water exchange, and the NG was collected as solid after freeze-drying.

### 2.9 PAH-PEG NG Coating

For His-tag decoration, PAH-PEG NGs were functionalized with COOH groups. This step was performed using COOH-PEG-COOH (Section 2.5). The latter (50 mg, 0.012 mmol) was dissolved in DDIW (6 mL), EDC (1.2 mg, 0.006 mmol) and NHS (0.72 mg, 0.006 mmol) were added, and the mixture was left under stirring for 30 min at RT. The molar ratio of COOH_PEG_:EDC:NHS was set equal to 2:1:1. The resulting activated PEG-NHS ester was added to a PAH-PEG NG solution (50 mg in 4 mL of DDIW) and the reaction left under stirring overnight at RT. Finally, the system was dialyzed against DDIW (MWCO = 12–14 kDa) for 2 days with daily water exchange and freeze-dried to collect the PEGylated NGs as sponge-like solid.

### 2.10 NG His-tag

The procedure for His-tag mimicking was performed on PAH-PEG and PAH-HA NGs and consisted of three steps: (i) conjugation of lys-NTA onto NG surface, (ii) metal complexation (Ni or Co),^39^ and (iii) His-tag of His-Rhod motifs.^40^

NGs (50 mg) were dissolved in PBS 1× (4 mL), and the residual COOH groups were activated by the agents EDC (46.5 mg, 0.3 mmol) and NHS (13.4 mg, 0.15 mmol) for 3 h under stirring. Then, a dialysis step in PBS (MWCO = 6–8 kDa) was performed for 12 h to remove the unreacted species and the byproducts; lys-NTA (16 mg, 0.06 mmol) was added to the system and left under stirring overnight at RT. The resulting suspension was dialyzed in DDIW (MWCO = 6–8 kDa) for 24 h and collected as dried powder after freeze-drying.

The resulting NGs were used for metal complexation. In detail, for Ni-based experiments, NGs (10 mg) were dispersed in TRIS buffer (pH = 9, 10 mL) and NiCl_2_ was added to reach a final concentration of 15 mM.

For Co-based tests, Co^3+^ salts were dissolved in PBS once to obtain a 2 mM solution, in which NGs were subsequently dispersed (final concentration 1 mg/mL).

In both cases, the final systems were stirred for 4 h at RT and dialyzed against PBS (pH = 7.4, MWCO = 6–8 kDa) for 24 h.

Finally, His-Rhod (37.37 mg, 0.06 mmol) was added to the purified solution of NG-lys-NTA-metal, using a 1:1 lys-NTA:His-Rhod molar ratio, and left under stirring at 4 °C overnight. Consequently, the product was dialyzed against DDIW, with daily water exchange, and freeze-dried.

Similarly, reactions with a molar excess of His-Rhod (i.e., lys-NTA:His-Rhod 1:10) were carried out to evaluate whether an increased availability of His residues could enhance metal-mediated tagging at the NTA sites.

### 2.11 Drug Encapsulation and Release

A stock solution of cisplatin (CIS) in DMSO (6 mg/mL) was prepared, and 150 μL were added to freeze-dried NGs (6 mg). After 10 min, to ensure the drug absorption within the meshes of the nanomaterials, 1.35 mL of DDIW was added to the NGs, to obtain a nominal mass ratio of 0.3 mg_cisplatin_/mg_NG_. The resulting suspension was vortexed for 1 min, and 100 μL was added to a VivaSpin filtration-dialysis chamber (MWCO = 100 kDa, Sartorius AG, Göttingen, Germany), against 900 μL of DDIW water as dialysis medium. The encapsulation efficiency (EE%), was determined as follows: withdrawn aliquots (3 × 100 μL) of the drug-loaded NGs were dried under air flow, redispersed in 0.9 mL of DDIW, filtered using a 0.22 μm PES filter, and analyzed via HPLC. EE% was calculated as reported in eq [Disp-formula ueq1]:

$$\mathrm{EE}\%=\frac{m_{\mathrm{cisplatin}}-m_{\mathrm{sol}}}{m_{\mathrm{cisplatin}}}\times 100$$ 1

where *m*_cisplatin_ is the total mass of added cisplatin and *m*_sol_ is the unloaded drug mass detected into the filtered aliquots. A drug calibration curve obtained via HPLC (λ = 230 nm, Figure S1) was used as a reference for the evaluation of the unloaded cisplatin. The drug loading (DL%) was estimated according to eq [Disp-formula ueq2]:

$$\mathrm{DL}\%=\frac{m_{\mathrm{cisplatin}}-m_{\mathrm{sol}}}{m_{\mathrm{NG}}}\times 100$$ 2

where *m*_NG_ is the mass of NGs.

The drug release profile from NG specimens was estimated at RT. Drug-loaded NGs were prepared as mentioned above and allowed to exchange against DDIW (VivaSpin system). At defined time points, the dialysis volume (900 μL) was withdrawn and replaced with fresh DDIW and analyzed via HPLC (λ = 230 nm). Based on the cisplatin calibration curve, the amount of released drug was estimated as (eq [Disp-formula ueq3]):

$$m_{\mathrm{rel}}\%(t)=\frac{m_{\mathrm{cisplatin},0}-m_{\mathrm{cisplatin},t}}{m_{\mathrm{cisplatin},0}}\times 100$$ 3

where *m*_rel_ % (*t*) is the percentage of drug released at time *t* and *m*_cisplatin,0_ and *m*_cisplatin,*t*_ are respectively the amounts of cisplatin present in the dialysis chamber at the starting point (*t =* 0, according to DL%) and at time *t*. Experimental data were collected from three independent replicates for each type of analyzed NGs and reported as the mean ± standard deviation (SD).

### 2.12 Grafting Density Evaluation

The amount of His-Rhod tagged to the nanoscaffolds was estimated via UV–vis spectrophotometry using a Tecan Spark (Tecan Group Ltd., Männedorf, CH), referring to its calibration curve (λ = 282, Figure S2). The amount of conjugated Rhod per mg of NG was defined as “grafting density”, and the investigated NG samples were prepared as follows: 1 mg of freeze-dried NGs was dissolved in DDIW and analyzed in triplicate, obtaining a value expressed in mg_tag_/mg_NG_, where mg_tag_ represents the quantity, in mass, of His-Rhod linked to the NGs.

### 2.13 Stability Assessment of Metal-His-Rhod Coordination

The rate of dissociation of His-Rhod from the NG specimens was analyzed through UV–vis spectrophotometry using a Tecan Spark (Tecan Group Ltd., Männedorf, CH), referring to its calibration curve (λ = 282 nm, Figure S2). NG samples were prepared as follows: 500 μg of freeze-dried NGs was dissolved in either PBS 1× (100 μL) or in EDTA solution (50 mM, 100 μL), as reported in Wegner at al.^41^ The NG solution was placed in the internal chamber of a VivaSpin (MWCO = 5 kDa) and dialyzed against PBS 1× and EDTA solution. Withdrawn aliquots were analyzed at specific time points from three independent replicas. Results are expressed in mg_tag–loss_/mg_tag_ [%], where mg_tag–loss_ represents the cumulative mass of His-Rhod dissociated from the NGs.

### 2.14 Characterization Techniques

#### 2.14.1 ^1^H NMR Analysis

Reaction products were analyzed by proton nuclear magnetic resonance (^1^H NMR) performed on a Bruker AC spectrometer (400 MHz, Bruker Corp., Billerica, USA) using D_2_O or CDCl_3_ as the solvent. All the samples were prepared at a concentration of 10 mg/mL. Each measurement was performed with 64 scans. A relaxation delay of 1.5 s was used for all samples, except for the NaCMP synthesis, for which a 5 s delay was applied. The chemical shifts were reported as δ values (ppm) with respect to trimethylsilylpropanoic acid (TMSP) or tetramethylsilane (TMS) as the internal standard.

#### 2.14.2 ATR-FTIR Analysis

Attenuated total reflectance Fourier transform infrared spectroscopy (ATR-FTIR) was performed using a Cary 630 spectrometer (Agilent Technologies Italia, Cernusco sul Naviglio, Italy). Spectra were acquired with 64 scans at a resolution of 2 cm^–1^ in the wavenumber range of 4000–650 cm^–1^ at RT.

#### 2.14.3 DLS and ELS Analyses

NG size, polydispersity, and ζ-potential were evaluated through dynamic light scattering (DLS) and electrophoretic light scattering (ELS) utilizing a Zetasizer Nano ZS (Malvern Instruments, Malvern, UK). Samples were dispersed in DDIW at a concentration of 0.1 mg/mL. Readings were performed in triplicate at 25 °C, and the average was taken as the representative value for each sample.

#### 2.14.4 Powder X-ray Diffractometry (XRD)

The effective presence of Co^3+^ and Ni^2+^ in the NG complexation was verified on a Bruker D2 Phaser X-ray diffractometer, using a Linxeye-2 (1D mode) detector, equipped with a Cu *K*α radiation (λ = 1.54184 Å). NGs after complexation were dialyzed in DDIW for 24 h (MWCO = 6–8 kDa) and freeze-dried. The resulting powder was placed on an aluminum holder, covered with a glass slide, and analyzed. IRD patterns were recorded over a 2θ range of 10–90°, with a step size of 0.01° and a scan time of 0.8 s per step.

#### 2.14.5 HPLC Analysis

Cisplatin release from the NGs was evaluated via high-performance liquid chromatography (HPLC) using an Agilent 1100 system (Agilent Technologies) equipped with a diode array detector set at λ = 230 nm. The separation was performed on an Agilent Eclipse C18 column (250 mm length, 4.6 mm inner diameter) under isocratic conditions using DDIW as the mobile phase. Analyzed samples consisted of aqueous aliquots (900 μL) withdrawn at defined time points during the drug release experiments performed in VivaSpin chambers (MWCO = 100 kDa).

### 2.15 Cell Culture

The human ovarian carcinoma cell line OVCAR3 (ATCC HTB-161) was obtained from the American Type Culture Collection (LGC Standards, Teddington, UK) and cultured in RPMI 1640 medium (1×), supplemented with GlutaMax Supplement (Cat# 61870036; Thermo Fisher Scientific), 20% fetal bovine serum (Cat# F524-500 ML; Merck KGaA), 10,000 U mL^–1^ penicillin, and 10 mg/mL streptomycin. The cells were incubated at 37 °C under a humidified atmosphere with 5% CO_2_.

### 2.16 NG Biocompatibility and MTT Assay

An evaluation of the potential cytotoxicity of the NGs was conducted through MTT assay. 6.5 × 10^3^ cells per cm^2^ were seeded into a 96-well plate and incubated for 24 h. Dried NGs were sterilized with UV light. A stock suspension at a concentration of 0.1 mg/mL was prepared, administered to OVCAR3 cells and incubated for 24 h, at 37 °C with 5% CO_2_. Then, the assay was performed, measuring the activity of mitochondrial dehydrogenases in living cells in terms of absorbance at λ = 570 nm (Tecan Spark), after 3 h of exposure to MTT solution (0.5 mg/mL) in PBS at 37 °C and 5% CO_2_. The experiments were performed in triplicate. Similarly, an *in vitro* evaluation of the therapeutic effect related to the administration of cisplatin-loaded NGs was performed at a NG concentration of 0.1 mg/mL loaded with sterile cisplatin solution and suspended in cell medium to obtain a final drug concentration of 10 μM. Drug-loaded NGs were administered to cells, and the MTT assay was performed after 24 h of incubation. A reference group consisting of cells treated with pristine (i.e., nonencapsulated) CIS at the same final concentration of 10 μM in culture medium was used to compare the outcomes of NG-mediated conditions with the free drug solution.

### 2.17 Flow Cytometric Analysis and Cytotoxic Activity of NGs

OVCAR3 cells (2 × 10^5^) were cultured in 24-well plates with medium (Ctr) or the indicated Cy5-labeled NGs (0.1 mg/mL) for 24 h. At the end of incubation, apoptosis was evaluated by staining cells with CellEvent Caspase-3/7 Green ReadyProbes Reagent (no. R37111, Thermo Fisher Scientific, Italy), and the percentage of Cy5-positive and caspase-3/7-positive cells was quantified by flow cytometry (FACScalibur, BD Biosciences, Mountain View, California). Data were analyzed with FlowJo 10.8.1 (BD Biosciences).

### 2.18 Confocal Microscopy Analysis

To analyze the endocytosis of Cy5-labeled NGs, OVCAR3 cells (4 × 10^4^) adhered overnight on coverslips were incubated for 24 h with Cy5-labeled NGs (0.1 mg/mL), while cells maintained in culture medium alone served as the control. After fixation with 3% paraformaldehyde, cells were permeabilized and analyzed by a 40× oil-immersion confocal objective (Zeiss LSM 900). Nuclei were stained with DAPI. The Cy5 fluorescence intensities of either surface-bound or endocytosed NGs were quantified using Fiji ImageJ software and expressed as the relative intensity.

### 2.19 Statistical Analysis

Data were analyzed using Prism ver. 10.5.0 (GraphPad Software, San Diego, California) and reported as the mean ± SD if not otherwise specified. The statistical significance, set at the 0.05 level, was evaluated through one-way analysis of variance (one-way ANOVA) followed by Tukey’s multiple comparison test.

## 3 Results

### 3.1 Polymer Functionalization and Characterization

The hydroxyl groups of PUL and PEG were modified with specific motifs (i.e., carboxyl groups and imidazole groups, respectively) to ensure the chemical cross-linking with PAH and form NGs (Figure 1a: NaCMP, Figure 1b: Im-PEG-Im) or to decorate the nanoscaffolds (Figure 1c: COOH-PEG-COOH). The polymers were characterized through ATR-FTIR and ^1^H NMR analyses, comparing the resulting spectra with the starting materials. Their synthesis route is reported in Figure 1a–c.

**Figure 1:**
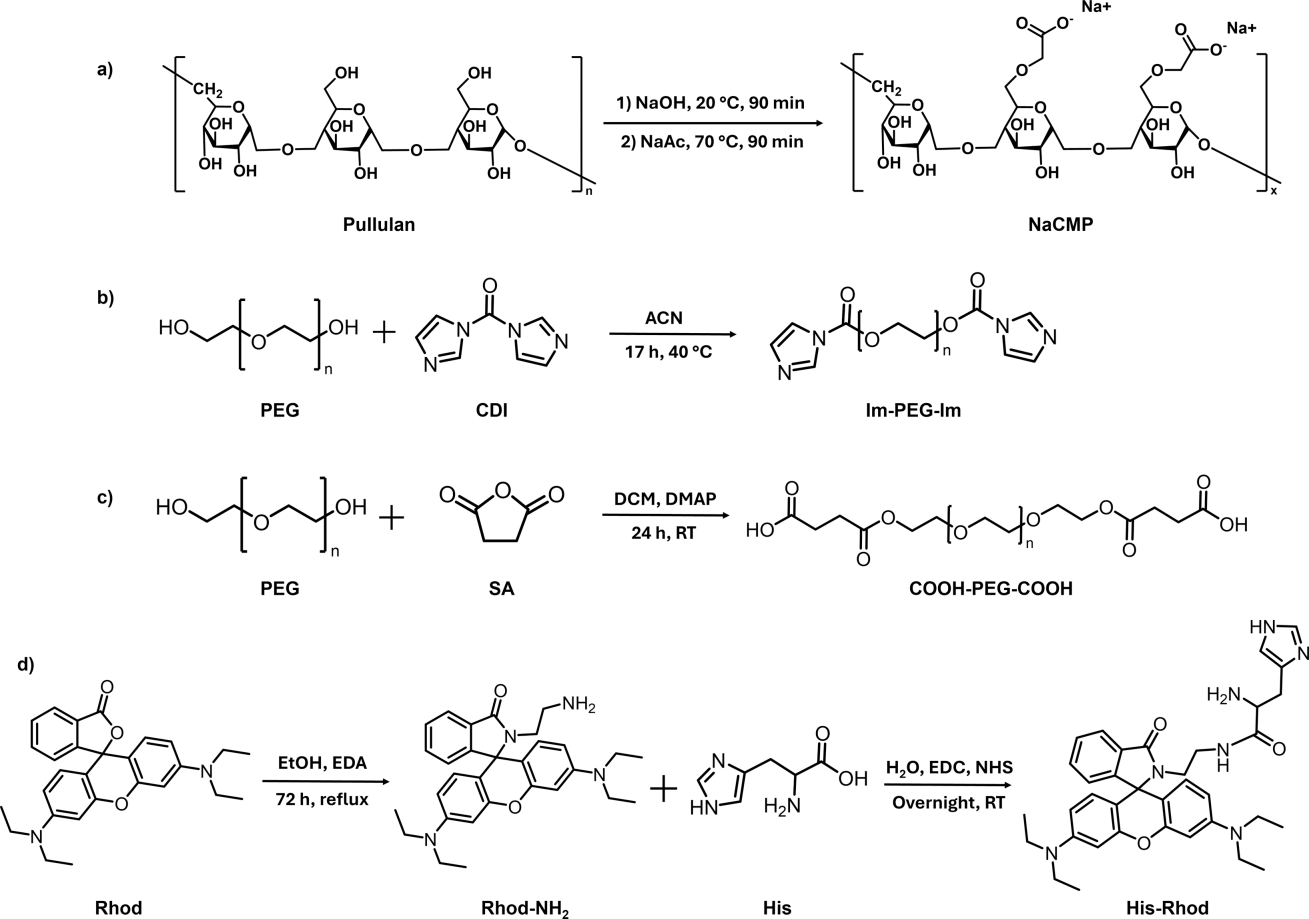
Synthesis route of the functionalization of polymers and Rhod for NG synthesis, decoration, and tagging: (a) carboxymethyl pullulan (NaCMP, representative substituted unit reported in the Figure), (b) Im-PEG-Im, (c) COOH-PEG-COOH, and (d) His-Rhod.

#### 3.1.1 NaCMP

The spectra of PUL and NaCMP were analyzed to confirm the carboxymethylation of the polysaccharide.

The ^1^H NMR spectra of PUL presented the typical signals^42,43^ of the polysaccharide, in particular the different peaks of the anhydroglucose unit between 3.4 and 4.2 ppm and those from the anomeric protons at 5.01 ppm (α-1,6 linkage) and 5.42 and 5.47 ppm (α-1,4 linkage). Regarding the ^1^H NMR spectrum of NaCMP, the new peaks between 4.1 and 4.6 ppm were related to the methylene protons of the substituent group. Therefore, the degree of substitution (DS) of NaCMP was assessed by peak area integration in its spectrum, as the ratio between the areas under the peaks in the range 4.1–4.6 ppm (corresponding to the methylene protons of the carboxymethylated group) divided by 2, and those of the anomeric protons in the 4.6–6.0 ppm region. The resulting DS was equal to 0.57 and referred to the anhydroglucose units of pullulan.^42^

Regarding ATR-FTIR, the typical signals of PUL were detected:^33,44^ the broad absorption band of the O–H stretching around 3284 cm^–1^, the C–H stretching around 2900 cm^–1^, the O–C–O stretching at 1633 cm^–1^, the C–O–H bending at 1346 cm^–1^, the C–O–C stretching at 1136 cm^–1^, and the characteristic signals of the α-1,6 linkage at 938 cm^–1^ and the α-1,4 linkage at 760 cm^–1^. In the NaCMP spectrum, the presence of new peaks corresponding to the C=O asymmetric stretching at 1597 cm^–1^ and bending at 1416 cm^–1^ provided evidence of the successful functionalization.^43^ Moreover, further confirmation of the DS of PUL hydroxyl groups was obtained by exploiting the chemical similarity between sodium carboxymethyl cellulose (NaCMC) and NaCMP, as previously discussed in literature for different cellulose derivatives.^45^ The ratio between the area under the asymmetric C=O stretching and that of the characteristic anhydroglucose unit (1188–802 cm^–1^) was calculated for NaCMP. By comparing this value with those obtained for three different sodium carboxymethilcellulose standards (Sigma-Aldrich), with known DS, i.e., 0.7, 0.9, and 1.2, a DS of 0.59 was determined for the functionalized polymer. This result was in close agreement with the value obtained from NMR analysis, confirming the consistency between the two techniques.

The ATR-FTIR and ^1^H NMR spectra of pullulan and its derivative are reported in Figure 2a and Figure 2b, respectively.

**Figure 2:**
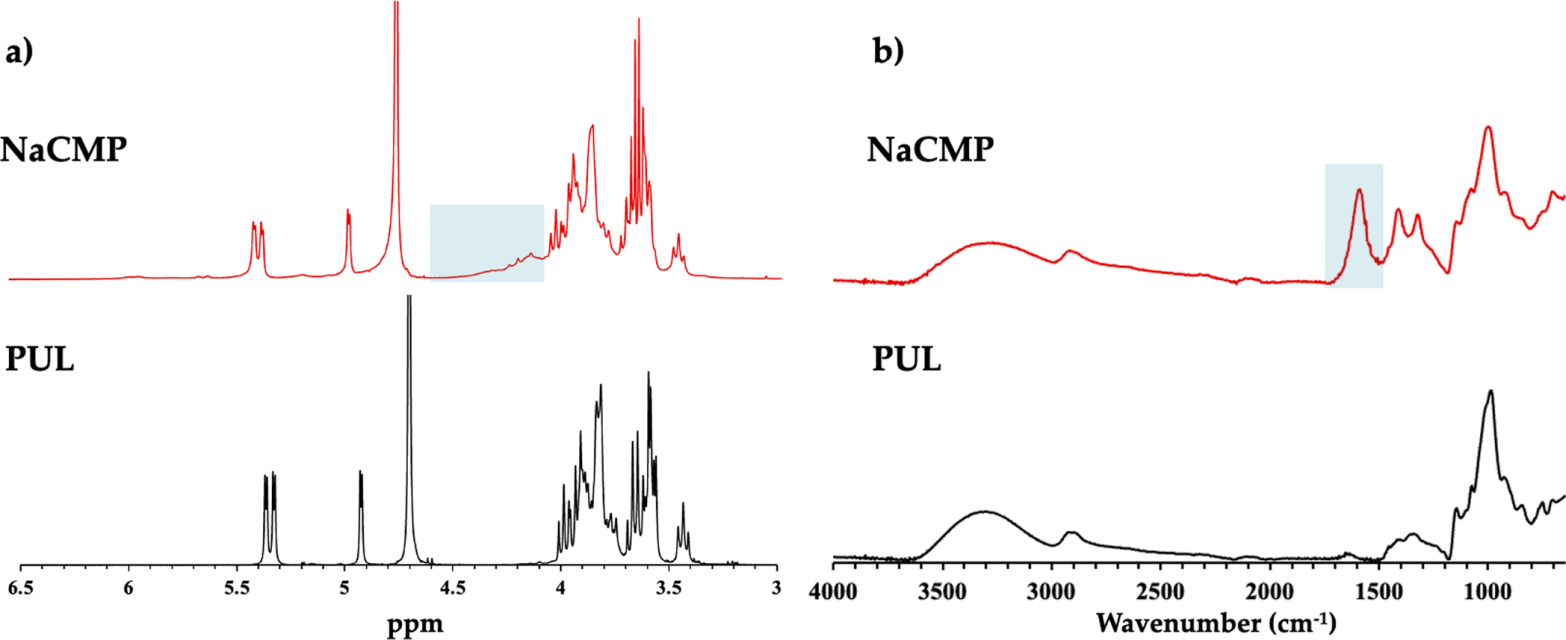
(a) ^1^H NMR analysis of PUL and NaCMP; the blue band highlights the methylene signals of the substituent groups in NaCMP. (b) ATR-FTIR analysis of PUL and NaCMP; the blue band highlights the C=O bonds in NaCMP.

#### 3.1.2 Im-PEG-Im

The ^1^H NMR spectrum of imidazole-activated PEG showed the characteristic peaks of the polymer backbone at 3.65 ppm, the terminal methylene protons −CH_2_–O– linking imidazole moieties at 4.32 ppm, and the imidazole peaks at 7.08, 7.61, and 8.33 ppm.^46^

ATR-FTIR presented the following PEG characteristic peaks: −OH stretching signal at 3400 cm^–1^, alkyl stretching at 2870 cm^–1^, and C–O–C stretching between 1058 and 1099 cm^–1^.^47^ Regarding the imidazole conjugation, characteristic signals of the functionalization can be identified at around 1760 cm^–1^, corresponding to the C=O stretching, and at 1970 cm^–1^, ascribable to the C=N stretching of the imidazole ring; the disappearance of the −OH stretching band further confirmed the successful activation of the polymer.

#### 3.1.3 COOH-PEG-COOH

Referring to PEG-diacid, the corresponding ^1^H NMR spectrum showed the multiplet signal of the methylene protons of the succinic derivative segment (2.55–2.65 ppm), characteristic peaks of PEG backbone in the range 3.4–3.9 ppm and the peak related to the methylene group adjacent to the ester linkage (4.25 ppm).^48^ In the ATR-FTIR spectrum (Figure S3a), the successful functionalization was highlighted by the peak at 1715 cm^–1^ corresponding to C=O stretching of the formed ester bond.

#### 3.1.4 His-Rhod

The effective synthesis of this intermediate was confirmed by ^1^H NMR and ATR-FTIR analyses. The ^1^H NMR spectrum reported the shift of the signal attributable to the methylene protons vicinal to the amide bond formed between Rhod-NH_2_ and His at 3.39 ppm (Figure S4). HPLC confirmed the successful reaction, highlighting the peaks of the product at λ = 282 and 555 nm, resulting in 89% purity. The ATR-FTIR spectrum (Figure S3b) showed the characteristic C=O stretching of the amide bond at 1686 cm^–1^ (peak not clearly detectable in the spectra of the Rhod-NH_2_ and His compound). Additionally, N–H stretching can be ascribed to the band at 3200–3400 cm^–1^, the amide band at 1590–1670 cm^–1^, and the C–N stretching at 1180 cm^–1^. This intermediate was used to define the calibration curve for the quantification of grafting density in NG samples.

### 3.2 NG Characterization

Synthesized NGs (Figure 3) were characterized via ^1^H NMR, ATR-FTIR, DLS, and ELS to confirm the chemical cross-linking and determine the hydrodynamic diameter (*D*_H_), polydispersity index (PDI), and ζ-potential. NG metal complexation was verified via XRD^49,50^ (Figure S5).

**Figure 3:**
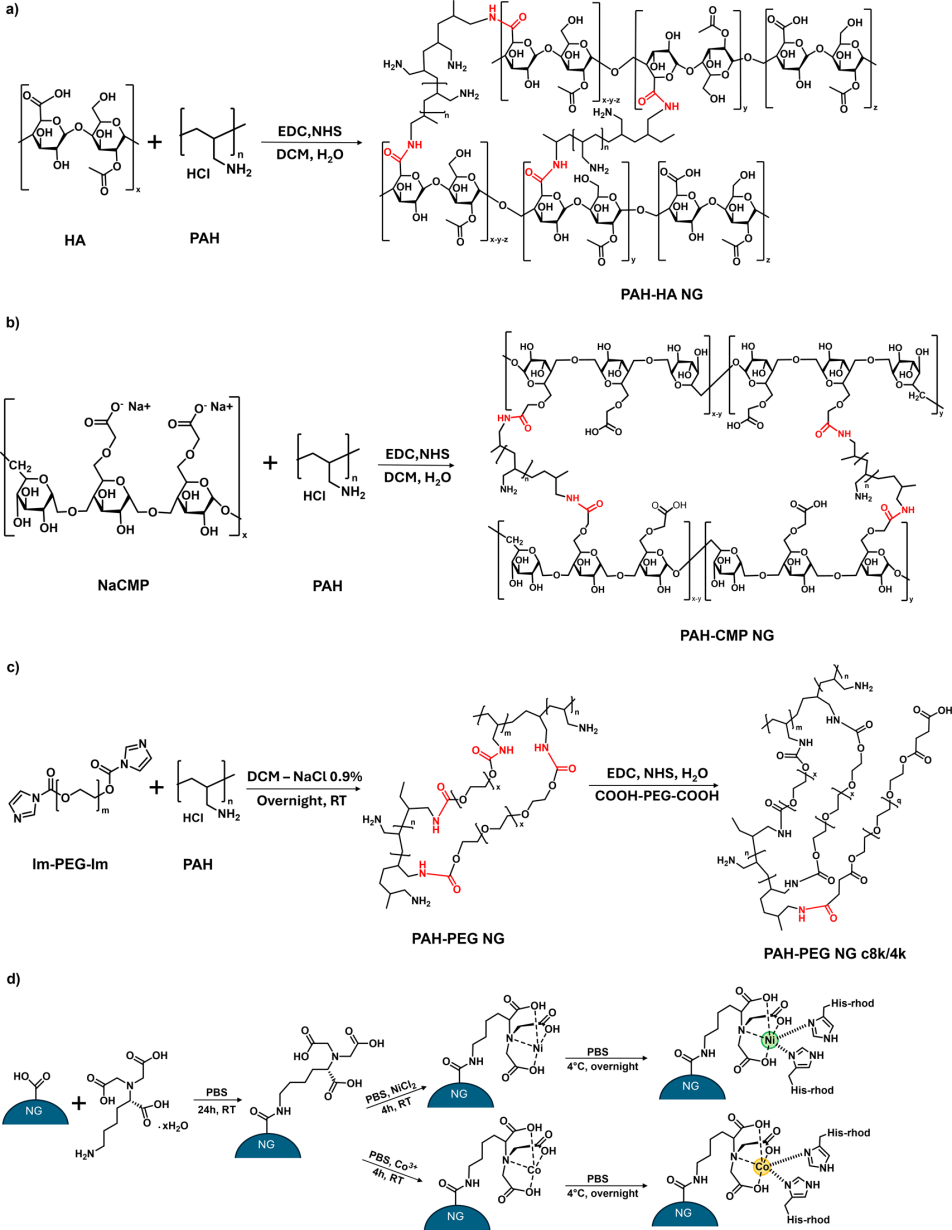
Synthetic scheme of analyzed NGs and representative His-tag functionalization: (a) PAH-HA NGs, (b) PAH-CMP NGs, (c) PAH-PEG NGs and surface PEGylation, and (d) His-Rhod conjugation through metal complexation (Ni^2+^ and Co^3+^). Newly formed linkages resulting from polymer cross-linking are highlighted in red.

The ^1^H NMR spectra of NGs showed a signal shift of the PAH methylene protons involved in the amide linkage between the two polymers, specifically from 2.40 to 3.40 ppm in the PAH-HA sample, from 2.45 to 3.51 ppm in PAH-CMP NGs, and from 2.40 to 3.50 ppm in PAH-PEG NGs (Figure 4a–c). Additionally, for PAH-PEG NGs, the disappearance of the imidazole group peaks (7.08–8.33 ppm) confirmed the successful synthesis route.

**Figure 4:**
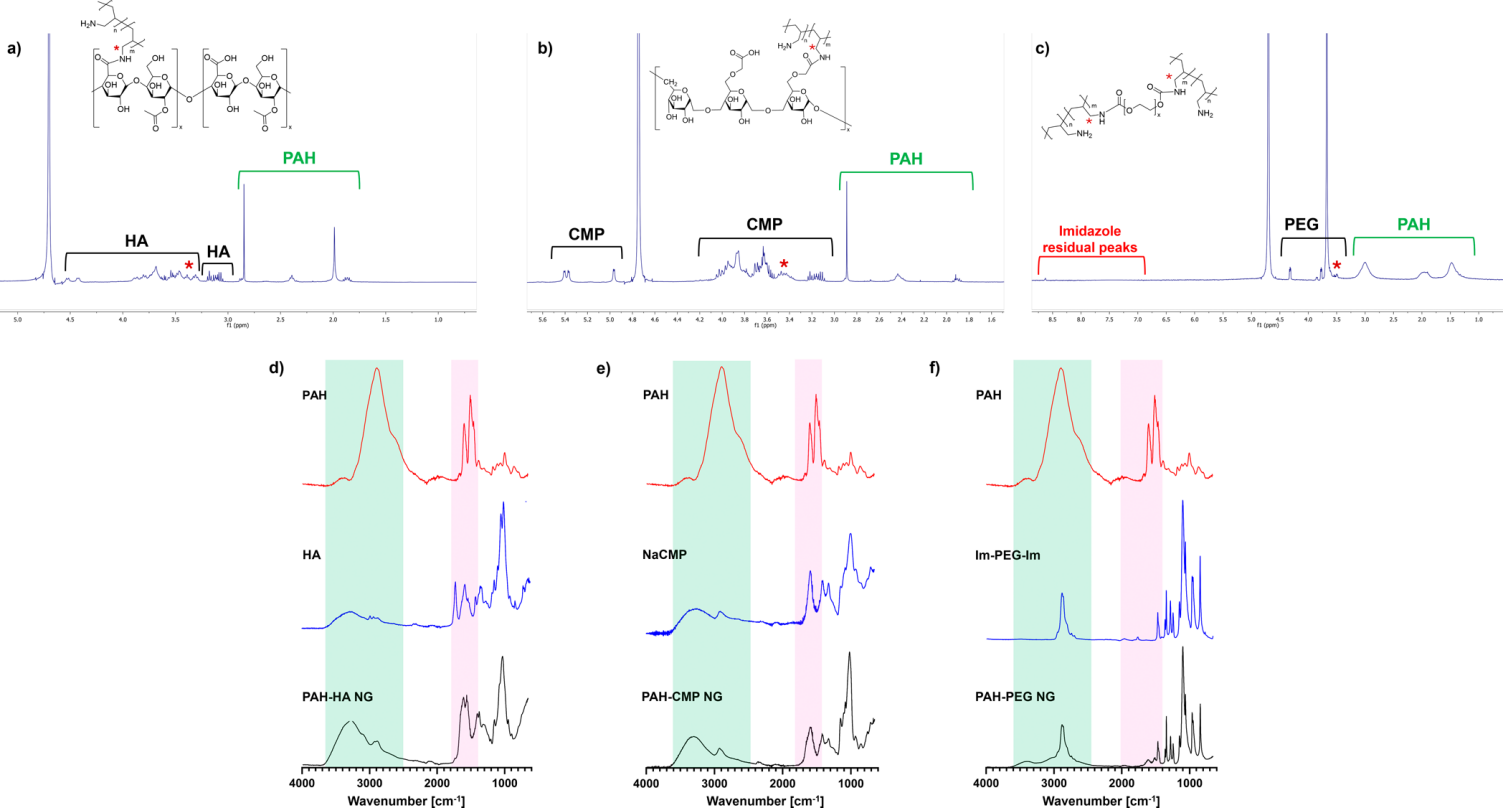
(a–c) ^1^H NMR spectra of synthesized NGs: PAH-HA NGs (a), PAH-CMP NGs (b), and PAH-PEG NGs (c). The signal shift of PAH methylene protons involved in the amide bond formation is marked with * (in red). (d–f) ATR-FTIR spectra of NGs compared to the starting polymers used in their formulation: PAH-HA NGs (d), PAH-CMP NGs (e), and PAH-PEG NGs (f).

Regarding ATR-FTIR analysis, the spectrum of PAH-HA NGs presented the characteristic bands of HA backbone, such as O–H stretching at 3270 cm^–1^, aliphatic C–H stretching at 2880 cm^–1^, carboxylic carbonyl stretching at 1680 cm^–1^, the bands of the C–O–C stretching, and the skeletal vibration involving the C–O stretching in the 1100–980 cm^–1^ range; PAH signals corresponding to the N–H stretching can be identified at ca. 3370 cm^–1^. The signal of C=O stretching at 1744 cm^–1^ can be ascribed to the formation of the amide bond confirming the formation of the nanoscaffolds (Figure 4d).

For PAH-CMP NGs, the ATR-FTIR analysis showed peaks ascribable to the d-glucosidic part in the range 757–932 cm^–1^, ether C–O stretching at 1087 cm^–1^, while successful NG formation was confirmed by the appearance of the amide C=O stretching band, observed as a modification of the CMP signal at 1620 cm^–1^, together with an increased intensity of the broad band between 3000 and 3500 cm^–1^, attributable to the O–H and N–H stretching vibrations of CMP and PAH, respectively (Figure 4e).

For PAH-PEG NGs, PAH-related N–H stretching can be identified around 3100–3400 cm^–1^, while the PEG characteristic bands are detectable at 2863 cm^–1^ (C–H stretching), 1460 cm^–1^ (C–H bending), and 1210–1090 cm^–1^ (C–O–C and C–O stretching). The peak at 1690 cm^–1^ can be ascribed to the C=O stretching of the formed carbamate linkage between the two polymers (Figure 4f).

NG size and ζ-potential were evaluated via DLS and ELS analyses; the results are reported in Table 1. PAH-CMP NGs showed larger dimensions compared with the other formulations, with a hydrodynamic diameter of 544 nm. According to the literature,^51,52^ these NGs exhibited dimensions exceeding the recommended threshold of ca. 300 nm, generally considered as the upper limit for a very efficient nanoscale drug delivery system based on soft polymeric materials. For this reason, PAH-CMP NGs were not further investigated. On the other side, PAH-HA and PAH-PEG specimens were characterized by sizes of 225 and 160 nm, respectively, and used for the His-tag conjugation.

**Table 1:** DLS and ELS Measurements of PAH-based NGs, Presenting Hydrodynamic Diameter (*D*_H_), Polydispersity Index (PDI), and ζ-Potential^a^

sample	*D*_H_ [nm]	PDI [−]	ζ-potential [mV]
PAH-HA	225.1	0.05	–33.7
PAH-CMP	544.2	0.08	–31.0
PAH-PEG	160.7	0.02	+40.3
PAH-PEG-c4k	238.9	0.06	+27.3
PAH-PEG-c8k	218.2	0.03	+3.4

a Measurements of *D*_H_, PDI, and ζ-potential were performed in triplicate, and the average value is reported.

The functionalization with His-Rhod required preliminary activation with lys-NTA, introducing chemical groups capable of metal ion complexation and coordination with His motifs. The lys-NTA was covalently bound to the nanoscaffold via amide coupling, involving the reaction between available carboxyl groups on the NGs and the amine group of lys-NTA.

However, PAH-PEG NGs inherently lack COOH groups. To introduce carboxyl functionalities, these NGs were decorated with COOH-PEG-COOH. The conjugation occurred between the residual amine groups of PAH and one terminal carboxyl group of PEG, resulting in the exposure of free carboxyl groups on the NG surface for subsequent His-binding steps. In particular, we coated NGs using two different PEG molecular weights: 4000 and 8000 g/mol. In both cases, successful grafting was achieved, leading to an increase in NG size, while maintaining dimensions within a suitable range for biological applications (i.e., <300 nm). Specifically, the hydrodynamic diameter measured 238.9 nm for PEG-4k-coated PAH-PEG NGs (hereinafter PAH-PEG-c4k) and 218.2 nm for PEG-8k-coated ones (PAH-PEG-c8k). The difference in size between the two decorated NGs could be ascribed to the different chain length-dependent entanglement and the relative packing density.^53^ High molecular weight might promote a “mushroom” conformational regime than a brush conformation, leading to a more compact NG system.^54,55^ This dual PEG coating strategy aimed to demonstrate the feasibility and general applicability of the His-tag conjugation procedure on this type of NG.

Regarding ζ-potential, PAH-HA NGs displayed a negative surface charge, which can be ascribed to the deprotonated HA carboxyl groups. Conversely, PAH-PEG NGs exhibited a positive ζ-potential, consistent with the presence of protonated amine groups from PAH. After PEGylation with COOH-PEG-COOH, these NGs maintained a positive charge, although with a decrease in magnitude. The reduction was more pronounced for the formulation with PEG-8k coating, likely due to the enhanced electrostatic shielding and surface coverage provided by longer PEG chains compared to those of the counterpart PEG-4k.

Moreover, to evaluate the impact of His-tag conjugation, the NG hydrodynamic diameter and ζ-potential were assessed after His-tag functionalization. Under aqueous and physiological conditions, NGs exhibited a slight increase in size (in the order of tens of nanometers, Table 2 and Supporting Information, Figure S6), consistent with the steric contribution arising from the functionalization. In all cases, the particle size remained below the typical threshold required for drug delivery and intracellular administration.

**Table 2:** DLS and ELS Measurements of His-Rhod Conjugated NGs, Presenting Hydrodynamic Diameter (*D*_H_), Polydispersity Index (PDI), and ζ-Potential^a^

sample	*D*_H_ [nm]	PDI [−]	ζ-potential [mV]
PAH-HA-Ni + His-Rhod	250.3	0.04	–11.8
PAH-PEG-c4k-Ni + His-Rhod	265.6	0.08	–13.5
PAH-PEG-c8k-Ni + His-Rhod	240.1	0.06	–1.5
PAH-HA-Co + His-Rhod	238.9	0.03	–11.4
PAH-PEG-c4k-Co + His-Rhod	275.5	0.02	–14.7
PAH-PEG-c8k-Co + His-Rhod	242.0	0.07	–1.7

a Measurements of *D*_H_, PDI, and ζ-potential were performed in triplicate, and the average value is reported.

Additionally, NGs demonstrated good colloidal stability over time, as confirmed by size measurements up to 48 h (Supporting Information, Figure S6). Regarding the ζ-potential, the results showed a decrease in net surface charge compared to the nonconjugated specimens (Table 2 and Supporting Information, Figure S7). This behavior could be attributed to the presence of His-Rhod on the NG external layer, which partially shielded the original surface charge.

Furthermore, TEM images (Supporting Information, Figure S8) revealed a quasi-spherical morphology of the decorated nanoscaffolds and confirmed the size differences among the samples, as observed by DLS analysis. In TEM, the slight reduction in the NG dimension could be attributed to the dry state of the specimens for this analysis, whereas DLS measurements were performed on fully solvated NGs, and thus reflect the hydrodynamic diameter. This discrepancy further supports the intrinsic swelling behavior of the nanogels in aqueous environments.

### 3.3 Grafting Density and Metal Coordination Stability

PAH-HA and decorated PAH-PEG NGs were then functionalized with lys-NTA, which acts as a chelator for metal ions through its three carboxylate groups and one amine nitrogen. The coordinated metal ion, in turn, binds to the imidazole groups of histidine residues in the His-tag. Both Ni^2+^ and Co^3+^ were tested to assess their efficiency and specificity in mediating His-tag interactions. The choice of metal ion can significantly affect the coordination strength, binding affinity, and selectivity, thereby influencing the overall performance of the functionalization. Ni^2+^ is commonly employed, especially in immobilized metal affinity chromatography, due to its strong affinity for histidine residues,^56^ while Co^3+^ has been reported to provide higher specificity and reduced nonspecific binding.^41^ Therefore, both metal ions were evaluated to identify the optimal conditions for stable and efficient His-tag conjugation. The effective linkage of Rhod to the NGs was confirmed by ATR-FTIR analysis (Supporting Information, Figure S3) and evaluated as grafting density (GD), and defined as the mass of His-Rhod per unit of mass of NGs. This parameter was calculated by referring to the calibration curve of the His-Rhod (Supporting Information, Figure S2), and the results are reported in Table 3.

**Table 3:** Results of Grafting Density (GD) Analysis Performed on NGs, Expressed in mg_tag_/mg_NG_ ^a^

NG sample	GD_Ni_ [mg_tag_/mg_NG_]	GD_Co_ [mg_tag_/mg_NG_]
PAH-HA	0.101	0.426
PAH-PEG-c4k	0.033	0.351
PAH-PEG-c8k	0.031	0.269

a Both the formulation with Ni^2+^ and Co^3+^ has been analyzed and indicated in the rows as GD_Ni_ and GD_Co_, respectively. The analysis has been performed on NGs reacted with His-Rhod tag in a molar ratio equal to 1:1 (lys-NTA:His-Rhod).

PAH-HA NGs exhibited a higher GD compared to their PEG-based counterparts, both in the presence of Ni^2+^ and Co^3+^. This difference could be attributed to the varying availability of carboxyl groups in the two NG formulations. Specifically, HA contains one −COOH group per monomeric unit, whereas in PAH-PEG NGs, carboxyl groups are introduced only via PEG coating and estimated as a free one per PEG macromolecule. Statistically, this implies a higher overall availability of carboxyl groups in PAH-HA NGs (despite the partial involvement of −COOH groups in nanoscaffold formation). Consequently, a more efficient lys-NTA conjugation occurred in PAH-HA NGs, leading to increased metal ion complexation and enhanced His-tag binding.

At the same time, referring to the decorated specimens, a different GD was observed under Co^3+^ conditions. This can be explained according to the molecular-weight-dependent surface distribution of the PEG-diacid chains, with PEG-4k causing less steric hindrance and thereby exposing more accessible −COOH groups for grafting onto the NGs.

Furthermore, in all cases, we observed a more efficient His-tag, up to one order of magnitude, using Co^3+^ vs Ni^2+^.

This outcome can be explained considering the higher thermodynamic and kinetic stability of Co^3+^ complexes compared to those of Ni^2+^ ones. In fact, Co^3+^ complexes have significantly higher formation constants than Ni^2+^ complexes in similar coordination environments and are exchange-inert, undergoing slow ligand exchange. This minimizes nonspecific interactions, preferring selective binding to the imidazole moieties of His.^41,57^ In contrast, Ni^2+^ sites show lower stability and a more reversible character, which can lead to reduced GD after purification, with partial loss of bound groups during the final dialysis step.

Similar considerations can be extended to the samples prepared with a lys-NTA:His-Rhod ratio of 1:10, whose GD is reported in the Supporting Information (Table S1).

To further support these considerations and experimentally assess the coordination stability of Co^3+^- and Ni^2+^-conjugated systems, His-Rhod release studies were performed under both physiological and challenging conditions. Specifically, the release of His-Rhod was monitored as a direct probe of metal-His coordination stability in PBS and EDTA solution (50 mM).

NGs in PBS (Figure 5a) showed no relevant His-Rhod release, with up to ca. 10% of Rhodamine detected over time. In particular, at equivalent NG formulation, Co-coordinated systems exhibited higher His-Rhod retention stability than the Ni counterparts, confirming the higher kinetic inertness of the cobalt-based complexation mechanism in a physiologically relevant medium.

**Figure 5:**
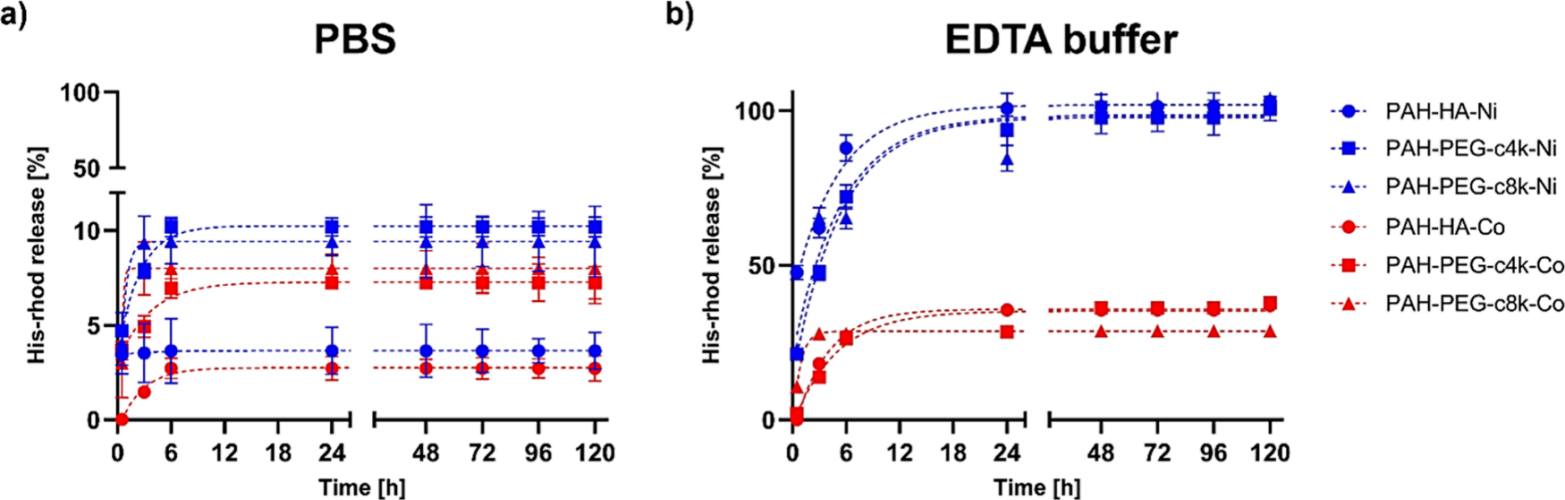
Metal-complex stability evaluation performed by analyzing His-Rhod release from Ni^2+^ (blue) and Co^3+^ (red) conjugated NGs, in (a) PBS and (b) EDTA buffer.

A more pronounced difference was observed under the EDTA conditions (Figure 5b). EDTA was selected as a challenging agent due to its strong chelating ability toward metal ions, enabling competitive binding to the metal–ligand complex. Moreover, its multidentate coordination behavior makes it a suitable competitor for NTA-like ligands present on the NGs. In this case, Co^3+^-conjugated nanoscaffolds maintained a significantly higher fraction of bound His-Rhod, whereas Ni^2+^-based systems showed a markedly faster release (completed in approximately 24 h). These results further confirmed the superior performance of Co^3+^ coordination compared to Ni^2+^.

Overall, these outcomes highlight the potential of Co^3+^ as an effective immobilizing agent for decorating nanocarriers with His-tagged motifs.

Furthermore, given the general applicability of the His-tag approach, these results may serve as a guideline for the conjugation of other His-bearing biomolecules, such as peptides or antibodies (Supporting Information, Figure S12), thus opening new avenues in the formulation of nanogels with highly selective inter- and intracellular interactions for advanced therapeutic strategies.

Given the higher grafting efficiency achieved for the His-Rhod-decorated NGs using Co^3+^, these formulations were further examined in drug release studies, cellular uptake, and possible therapeutic effects.

### 3.4 Drug Release Evaluation

PAH-HA NGs, the PEGylated NG formulations, and the corresponding His-Rhod conjugated specimens using Co^3+^ were tested as the delivery nanosystem of cisplatin, a widespread drug used in chemotherapeutic treatments.^58^ Drug encapsulation was performed by exploiting the swelling behavior of NGs in the transition dried-hydrated state (i.e., sponge-like drug loading); in this way, the drug was absorbed within the nanomeshes. The EE% and DL% were estimated via HPLC analyses, reported in Table 4.

**Table 4:** Encapsulation Efficiency (EE%), Drug Loading Content (DL%), and Maximum Drug Release (*m*_rel_%) of the Analyzed NGs, Evaluated after 19 days (456 h)^a^

NG sample	EE%	DL%	*m*_rel_%
PAH-HA	99.02	14.85	64.19
PAH-PEG-c4k	97.78	14.67	74.61
PAH-PEG-c8k	95.04	14.26	77.84
PAH-HA+His-Rhod	80.46	12.07	93.43
PAH-PEG-c4k+His-Rhod	78.81	11.82	74.17
PAH-PEG-c8k+His-Rhod	76.47	11.47	85.36

a All the data were performed in triplicate and analyzed *via* HPLC at λ = 230 nm.

The three reference NG formulations (i.e., PAH-HA, PAH-PEG-c4k, and PAH-PEG-c8k) presented comparable EE% and DL% values. Regarding the drug release performances (Table 4 and Figure 6a), more noticeable variations were observed. In particular, *m*_rel_% was different between the HA-containing NGs and the PEG-based ones. This suggested a different drug–polymer interaction within the nanoscaffolds. We hypothesized that fewer physical interactions occurred between the PEG and CIS ligands in the PAH-PEG specimens. As a result, despite the presence of a PEG coating layer, the drug release was faster and not significantly affected by the molecular weight of the PEG-diacid.^59^ The incomplete drug release observed within the investigated time frame might be due to the interactions between CIS and the residual PAH primary amine groups and the PEG-COOH groups. In physiological conditions (pH 7.4), chlorine ligands and the Pt center could undergo ligand exchange with PAH, possibly leading to conjugate formation.^60,61^

**Figure 6:**
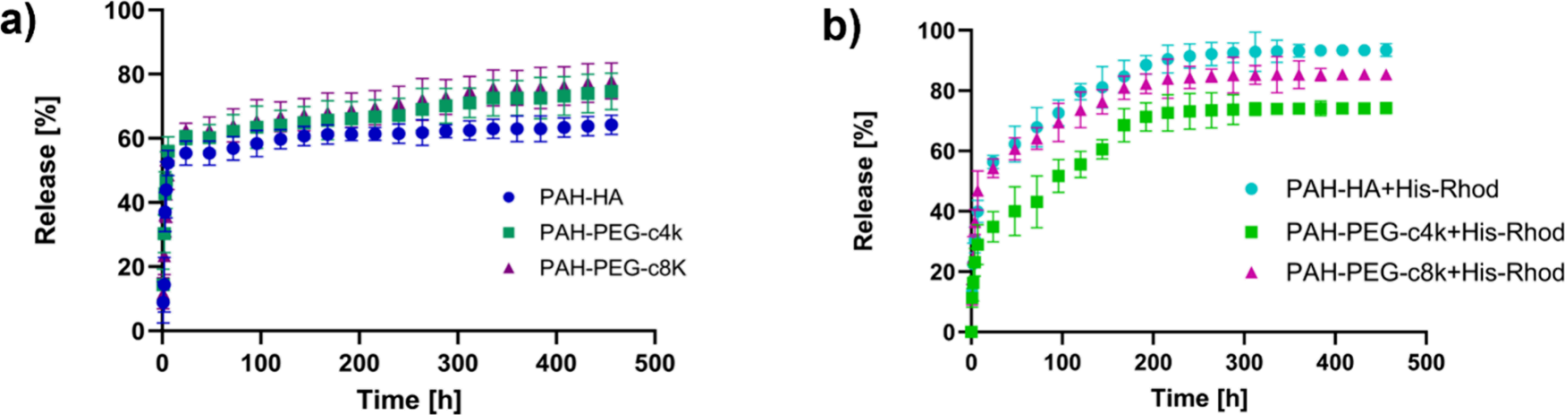
Cisplatin release profiles from NGs: (a) undecorated PAH-HA, PAH-PEG-c4k, and PAH-PEG-c8k NGs (i.e., without His-Rhod conjugation) and (b) His-Rhod-decorated counterparts obtained through Co^3+^ complexation.

On the other side, in PAH-HA NGs, additional interactions might also occur between cisplatin and HA residual carboxyl functional groups, delaying the release due to complexation effects.^62,63^ This resulted in a partial release of the loaded drug, reaching approximately 64% at 456 h.

The drug release from our NGs was consistent with previously reported HA- and amino-based cisplatin nanocarriers, which generally show prolonged and tunable release due to chelating ligand–metal or complexation effects.^64,65^

Referring to the drug release profiles (Figure 6), a common initial trend was observed across all of the samples, characterized by a faster release within the first 24 h, followed by a more gradual and sustained release over time, eventually reaching a plateau-like behavior (Figure 6a). This release pattern, commonly detectable in NG formulations, can be explained by a first quick diffusion of drug molecules localized within the outer regions of the nanomatrix. Subsequently, the drug distributed in the NG inner network diffuses through the polymeric chains and gradually releases into the surrounding medium.^66,67^

For His-Rhod grafted NGs, an increased and more sustained release of CIS was observed in PAH-HA+His-Rhod NGs, reaching 93% of the released drug. For this NG formulation, this result could be explained considering the reduction of residual free carboxyl groups in the nanonetwork due to the linkage with lys-NTA: this could decrease the drug complexation effects, leading to an overall higher amount of released CIS.

For the PEG-based NGs, the *m*_rel_% increase in PAH-PEG-c8k sample, compared to the corresponding reference without His-Rhod, could be correlated with the relevant decrease of free PEG carboxyl groups (involved in the amide linkage with His-Rhod), which minimized the CIS-PEG interaction. Nonetheless, the CIS-PAH interactions were preserved.

At the same time, a more gradual drug release during the first 100 h can be noticed compared to the trends in Figure 6a. This suggested that His-Rhod conjugation introduced additional steric interactions with the drug molecules diffusing through the NG, which could slow the initial drug diffusion and produce a more sustained release profile over time.

### 3.5 Flow Cytometric Analysis and NG Cellular Uptake

To validate the proposed NG specimens in a potential bio-related application, NGs were administered to OVCAR3 cells, a representative high-grade serous ovarian cancer cell line.^68^

NG engulfment by cells and their cytotoxic effects were investigated. OVCAR3 cells were cultured for 24 h with medium alone (Ctr) or with the indicated NGs. PAH-HA and PAH-PEG NG uptake and apoptosis activation were assessed by flow cytometry by measuring the percentage of Cy5-positive cells and caspase-3/7 (Casp3/7)-positive cells, respectively (Figure 7a,b). This evaluation, therefore, enables the implementation of a screening process that is based on the assessment of cellular uptake and the degree of toxicity.

Data in Figure 7a,b show that the neat NG formulations (i.e., PAH-HA and PAH-PEG specimens) were significantly engulfed by the OVCAR3 cells, without inducing detectable cytotoxic activity, as confirmed by the absence of increased Casp3/7-positive cells compared with the control. Regarding His-Rhod-grafted NGs, similar results were obtained for HA-based formulations using Co^3+^, showing uptake levels above 85% and Casp3/7-positive cells below 18%. This suggested that the surface decoration did not significantly affect NG internalization, indicating the potential for designing NGs enabling selective intracellular interactions, when the His-Rhod probe is replaced by appropriate functional biomolecules (e.g., peptides, enzyme substrates, or ligands) able to engage with intracellular pathways.

**Figure 7:**
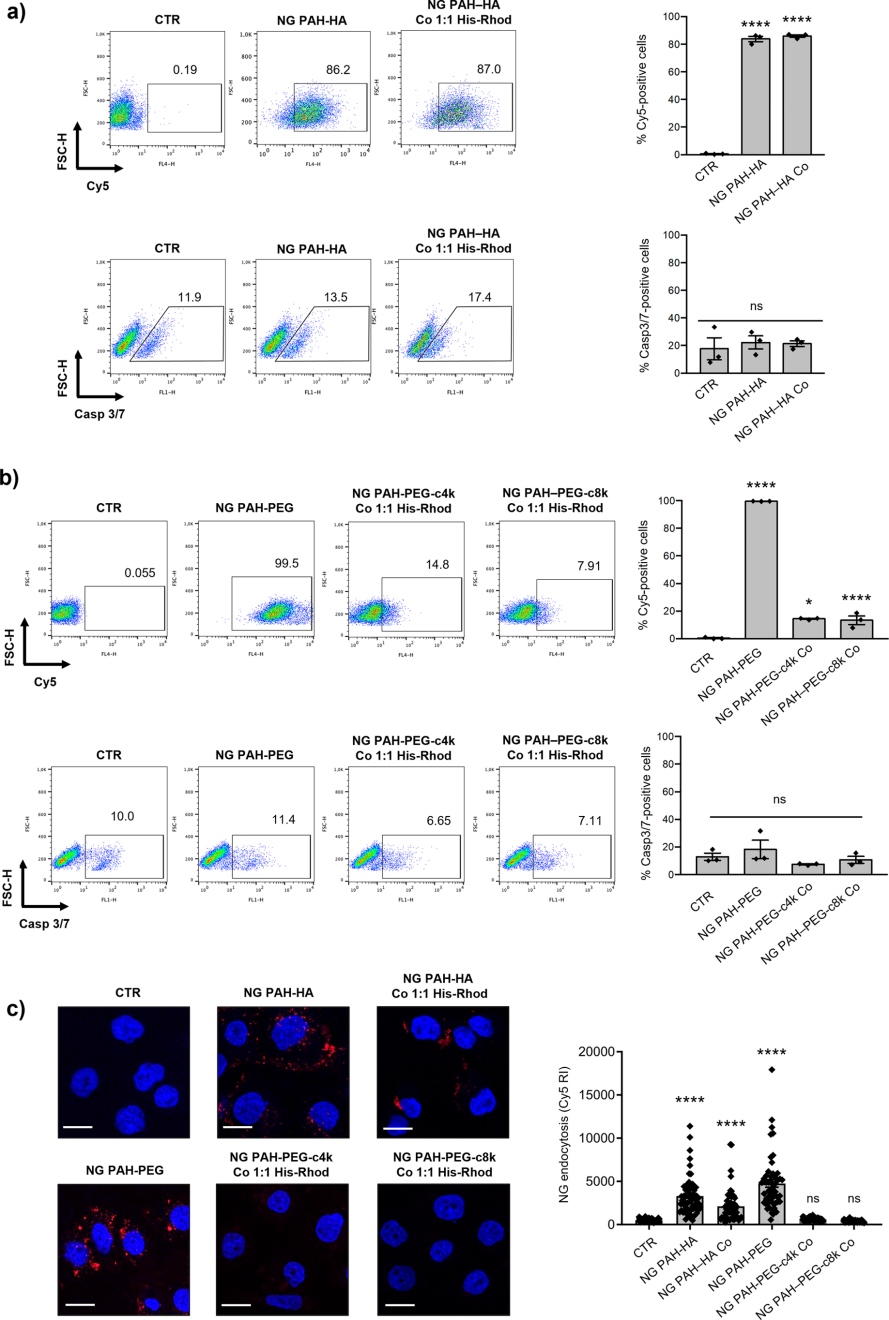
(a, b) NG uptake and apoptosis activation in OVCAR3 cells following the administration of the (a) PAH-HA NGs and (b) PAH-PEG NGs. OVCAR3 cells were incubated for 24 h with medium (CTR) or the indicated Cy5-labeled NGs (0.1 mg/mL) with a 1:1 lys-NTA:His-Rhod molar ratio tag functionalization. At the end of incubation, NG engulfment and caspase-3/7 (Casp3/7) activation were analyzed by flow cytometry. The percentages of Cy5-positive cells and Casp3/7-positive cells were calculated. (c) OVCAR3 cells incubated for 24 h with culture medium (CTR) or the indicated Cy5-labeled NGs. At the end of incubation, the endocytosis of NGs was analyzed by confocal microscopy. Scale bar = 20 μm. Nuclei were stained with DAPI (blue). Relative intensity (RI) of Cy5-positive NGs was quantified in at least 51 cells. Data represent mean ± SEM of triplicate cultures. Statistical significance was determined by Student’s *t* test. (*) *p* < 0.05, (**) *p* < 0.01, (***) *p* < 0.001, (****) *p* < 0.0001, ns = not significant.

On the other hand, His-tag modification of PEG-based NGs markedly reduced internalization (approximately 15–6.5%) without affecting cell viability (Figure 7b): This could be ascribed to the multistep decoration procedure involving PEG decoration, lys-NTA linkage, and subsequent metal complexation. PEGylation likely introduced steric hindrance, limiting interactions between the nanoscaffold and the cell membrane.^69,70^ Additionally, in this case, the resulting negative/near-neutral ζ-potential could contribute to reducing nonspecific interactions with the cell membrane.

This behavior suggested that His-tag-functionalized PEG-based NGs (where His-Rhod acts as a model biomotif) may be better suited for surface-level or receptor-mediated targeting (Supporting Information, Figure S12) rather than intracellular delivery.

For NGs decorated with a lys-NTA:His-Rhod ratio of 1:10, PAH-HA NGs showed higher cytotoxicity (Supporting Information, Figure S11 and Table S1). Following the NG uptake by cells, this could be attributed to the high Rhod content per NG, which could exert cytotoxic effects, within a specific concentration range, due to its chemical modification.^71^ Conversely, the PAH-PEG counterpart did not induce increased cytotoxicity, consistent with the low cell uptake, similarly to the lys-NTA:His-Rhod ratio of 1:1 formulation. Additionally, the percentage of Casp3/7-positive cells was lower than 10%.

To provide a full overview, flow cytometry was also performed on Ni-complexed NGs, showing trends similar to those observed for the Co-prepared specimens (Supporting Information Figures S9–S11).

The endocytosis of the decorated NGs was further validated by confocal microscopy. Consistent with the flow cytometry analysis, neat PAH-HA and PAH-PEG formulations, as well as the His-Rhod HA-based nanoscaffolds, were significantly engulfed by the OVCAR3 cells. In contrast, no significant internalization of His-tag-modified PEG-based NGs was observed (Figure 7c).

### 3.6 Effect of Drug Release on OVCAR3 Cells

A preliminary validation of the metabolic response of ovarian cancer cells to the administration of the reference NGs (i.e., PAH-HA, PAH-PEG-c4k, and PAH-PEG-c8k), their Co-mediated His-Rhod-coated counterparts, and the drug-loaded nanoscaffolds was conducted via MTT assay, after 24 h of incubation (Figure 8a,b). The obtained results showed no cytotoxic effects for all NG formulations, with only a slight reduction in cell viability observed in PAH-PEG-c8k NGs decorated with His-Rhod (*p* < 0.01 vs Ctr, although the material cannot be considered cytotoxic). Regarding drug-loaded NGs, the specimens without His-Rhod decoration (Figure 8a) exhibited a comparable effect to the administration of the drug in nonencapsulated form after 24 h. On the other side, coated NGs encapsulating CIS (Figure 8b) provided a higher therapeutic effect on cells. In detail, the administration of PAH-HA specimens resulted in a residual cell viability of ca. 15%, whereas for PAH-PEG samples, cell viability decreased to 38% for His-Rhod-coated PAH-PEG-c4k and 25% for the PAH-PEG-c8k counterpart. In all cases, these NGs outperformed the therapeutic effects of the administration of free CIS (*p* < 0.0001). The enhanced effect can be correlated with the increased drug release compared with the undecorated NGs (Figure 6a,b), and, in the case of the PAH-HA formulation, also to its higher cellular uptake,^72^ as confirmed by flow cytometry.

**Figure 8:**
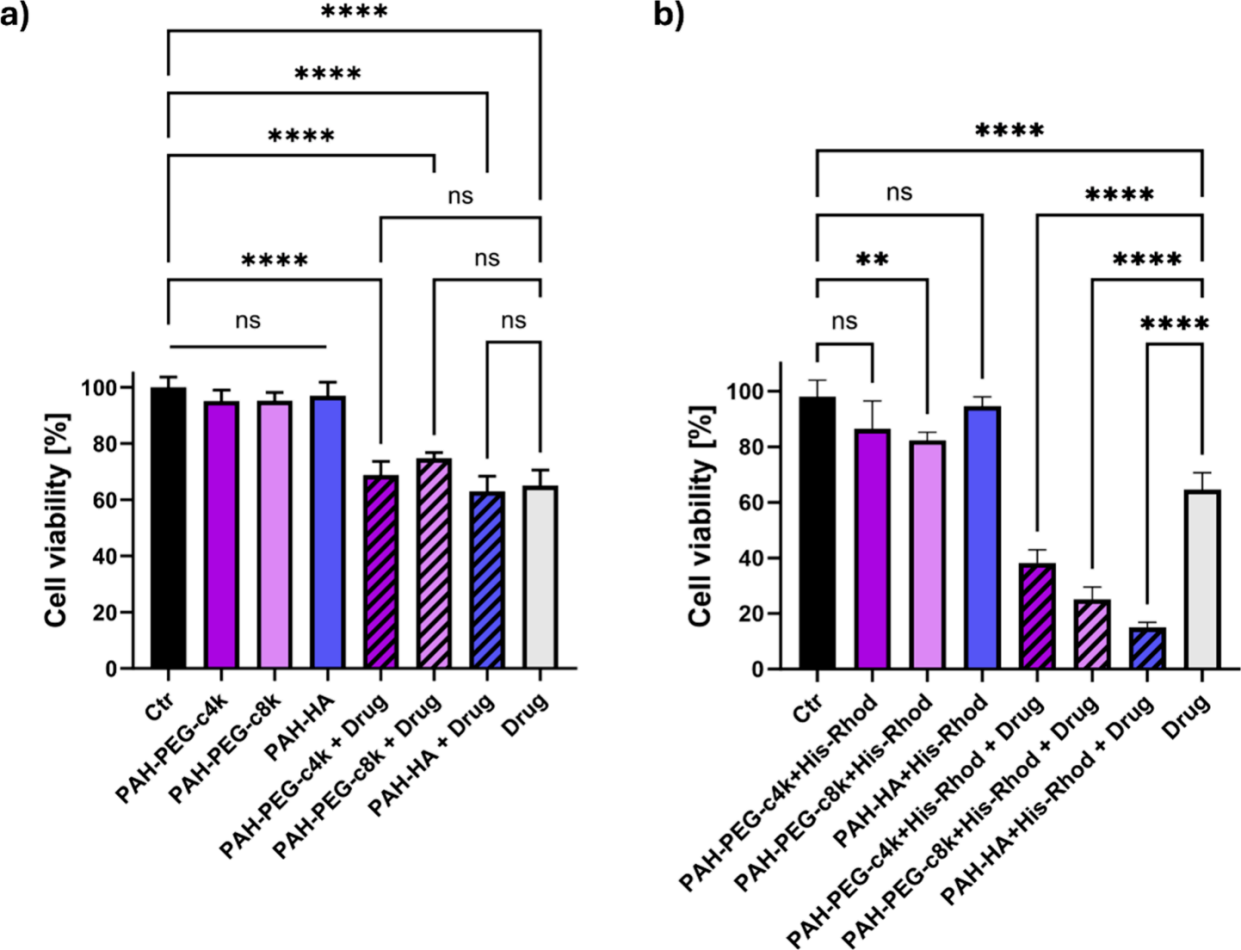
(a, b) Cisplatin (10 μM) induced cytotoxicity in OVCAR3 cancer cells at 24 h. In all groups, the therapeutic effects are expressed in terms of cell viability levels normalized against their internal controls (i.e., untreated cells, Ctr) measured through the MTT assay: (a) evaluation on empty and drug-loaded NGs without His-Rhod decoration; (b) effect of empty and drug-loaded NGs with Co^3+^-mediated His-Rhod conjugation. The results are reported as the mean ± SD of three independent experiments. Statistical analysis was performed using one-way ANOVA: (*) *p* < 0.05, (**) *p* < 0.01, (***) *p* < 0.001, (****) *p* < 0.0001, ns = not significant.

Overall, these findings highlighted the potential of His-Rhod-decorated NGs for drug delivery purposes, offering the opportunity to combine targeting capability imparted by the His-tag modification with a controlled and sustained drug release performance.

Based on these *in vitro* results, further *in vivo* studies will be necessary to validate the performance of His-tag-decorated NGs in physiological environments. Nevertheless, based on the coordination chemistry of His-tag systems, Co^3+^-mediated complexes are expected to provide enhanced stability of surface-conjugated ligands compared to Ni^2+^-based counterparts, potentially translating into improved circulation stability and reduced premature ligand displacement *in vivo.* Indeed, Co^3+^-NTA-His complexes display remarkable kinetic inertness, with half-lives of several days even in the presence of imidazole or strong chelators and under reducing conditions, in contrast to conventional Ni^2+^-NTA systems.^41,73^ Consequently, premature metal ion release into the bloodstream is anticipated to be limited for Co^3+^-coordinated NGs, whereas Ni^2+^-based coordination may be more susceptible to ligand exchange *in vivo*, particularly in chelator-rich or acidic compartments.^22,73^

## 4 Conclusions

This study successfully developed polyallylamine-based NGs decorated with representative His-tag motifs through metal coordination chemistry. In particular, the strategy was validated using two different metal ions: Ni^2+^, conventionally used in coordination-based systems, and Co^3+^, here proposed as an alternative and innovative approach for this purpose.

Notably, Co^3+^ exhibited superior performance compared to Ni^2+^, achieving a higher His-tag grafting density, likely due to its favorable ligand exchange properties and higher thermodynamic and kinetic stability yet irreversible complexes formation.

This work demonstrates the potential of cobalt-mediated His-tag conjugation as an efficient, noncovalent approach for decorating polymer-based NGs, overcoming the limitations of conventional covalent coupling that could compromise ligand functionality. Additionally, this provides the first experimental evidence of NG cobalt-based complexation as a viable route for surface decoration and targeted functionality.

The resulting NGs also exhibited a controlled cisplatin release profile in ovarian cancer cells, confirming their applicability for targeted therapeutic treatments, outperforming the effect of administration of the drug in free form.

Overall, the proposed strategy expands the toolkit for NG decoration, offering a versatile and effective route to conjugate His-tagged biomolecules. This approach opens new perspectives for the rational design of actively targeted nanotherapeutics with enhanced selectivity and reduced systemic toxicity.

## Supplementary Material



## Notes

Calibration curve of cisplatin; calibration curve for His-Rhod; results of grafting density (GD) analysis performed on NGs; ATR-FTIR spectra of COOH-PEG-COOH, His-Rhod, PAH-HA NG tagged with His-Rhod using Co^3+^, PAH-PEG NGs tagged with His-Rhod using Co^3+^; ^1^H NMR spectrum of His-Rhod; XRD characterization of NGs after metal complexation; colloidal stability of NGs; TEM images of NGs; flow cytometry of OVCAR3 with NGs; and 6xHis complexation (PDF)
