# Supplementary material for: Metal-Coordinated His-Tag Functionalization of Polymeric Nanogels for Therapeutic Applications
Source: ACS Appl Nano Mater. 2026 Mar 12;9(12):5309–25. doi: 10.1021/acsanm.5c05531 (PMC13073731; doi:10.1021/acsanm.5c05531)
Supplement: Supplementary file 1 [file an5c05531_si_001.pdf]

# Supporting Information

## Metal-Coordinated His-Tag Functionalization of Polymeric Nanogels for Therapeutic Applications

*Camillo Colli*[a,†], *Andrea De Filippis*[a,†], *Gabriele Galbo*[a], *Martina Kunkl*[b], *Julia Ivanova*[c], *Susan Fornoni*[a], *Joachim P. Spatz*[c,d,e], *Laura Rosanò*[f], *Davide Moscatelli*[a], *Loretta Tuosto*[b], *Emanuele Mauri*[a*]

[a] Department of Chemistry, Materials and Chemical Engineering "Giulio Natta", Politecnico di Milano, piazza Leonardo Da Vinci 32, 20133 Milan, Italy

[b] Department of Biology and Biotechnologies Charles Darwin, Istituto Pasteur Italia-Fondazione Cenci Bolognetti, Sapienza University of Rome, Rome 00185, Italy

[c] Max Planck Institute for Medical Research, Bildungscampus Heilbronn, Dept. of Cellular Biophysics, 74076 Heilbronn, Germany.

[d] Institute for Molecular Systems Engineering and Advanced Materials, Heidelberg University, 69120 Heidelberg, Germany.

[e] Max Planck School Matter to Life, Bildungscampus Heilbronn, 74076 Heilbronn, Germany.

[f] Institute of Molecular Biology and Pathology, National Research Council, Via degli Apuli 4, Rome, 00185, Italy

*corresponding author Tel.: +39 0223993397; e-mail: emanuele.mauri@polimi.it

† equally contributed to this work

*Cisplatin calibration curve*

Figure S1 shows the calibration curve of cisplatin used for the estimation of NG drug loading and release. The calibration curve was obtained by analyzing a series of cisplatin solutions through HPLC (λ = 230 nm), starting from a 1 mg/mL stock solution and preparing serial dilutions down to 100 ng/mL.

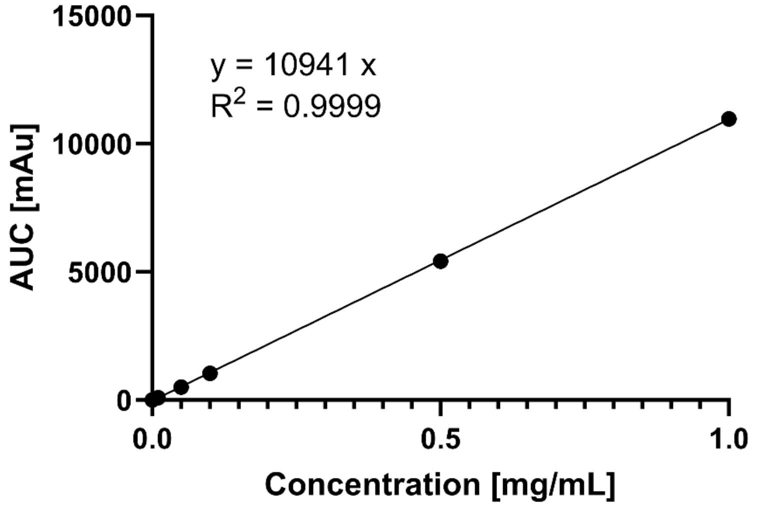


**Figure S1.** Calibration curve of cisplatin (λ = 230 nm).

*His-Rhod calibration curve*

His-Rhod was dissolved in demineralized water at a concentration of 0.5 mg/mL and subsequently diluted in a series of solution up to 1 μg/mL The absorbance of each solution was measured by UV–vis spectrophotometry at λ = 282 nm. Optical density (OD) values were plotted against His-Rhod concentrations to generate the calibration curve (Figure S2), which was then used to estimate the grafting density (GD) of His-Rhod on the NGs.

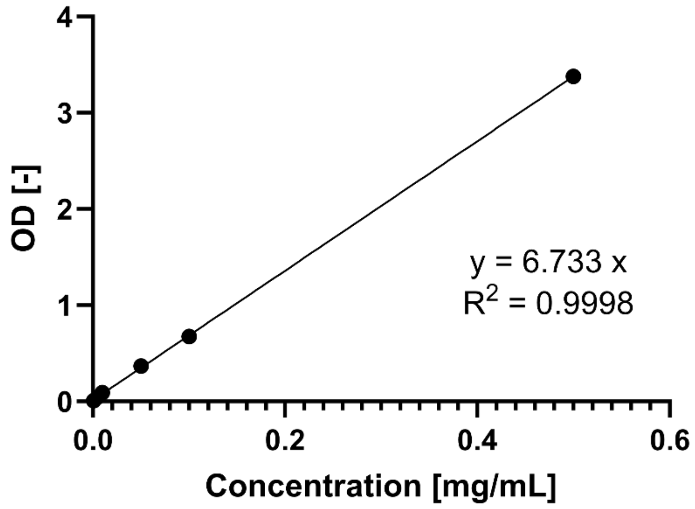


**Figure S2.** Calibration curve for His-Rhod (λ = 282 nm).

*NG Grafting Density for lys-NTA:His-Rhod 1:10 molar ratio*

Table S1 reports the GD values obtained using a lys-NTA:His-Rhod molar ratio of 1:10 for the His-tag conjugation. Compared to the 1:1 ratio, a 1.5-fold increase in GD was observed for PAH-HA NGs, while only a slight increase was detected for PAH-PEG specimens. This suggested that, for PEG-based NGs, the His-Rhod content approached the maximum level of conjugation already at the 1:1 ratio.

**Table S1.** Results of grafting density (GD) analysis performed on NGs, expressed in $mg_{tag}/mg_{NG}$, using a molar ration lys-NTA:His-Rhod equal to 1:10. Both the formulation with $Ni^{2+}$ and $Co^{3+}$ have been analyzed and indicated in the rows as $GD_{Ni}$ and $GD_{Co}$ respectively.

| NG Sample | $GD_{Ni}$ [$mg_{his}/mg_{NG}$] | $GD_{Co}$ [$mg_{his}/mg_{NG}$] |
|---|---|---|
| PAH-HA | 0.111 | 0.653 |
| PAH-PEG-c4k | 0.037 | 0.389 |
| PAH-PEG-c8k | 0.033 | 0.285 |

*ATR FT-IR spectra of diacid PEG, His-Rhod and His-Rhod conjugated NGs*

Figure S3 shows the ATR-FTIR spectra of COOH-PEG-COOH, His-Rhod, and the Cobalt-mediated His-Rhod tag of NGs. In particular, in His-Rhod spectrum (Figure S3b) the N-H stretching band is detectable in the range 3400-3200 $cm^{-1}$, C=O stretching of the formed amide bond between amine-Rhod and histidine at 1730 $cm^{-1}$ and the imidazole bands can be ascribed to the peaks in the range 1605-1500 $cm^{-1}$. The His-tag conjugation in NG spectra can be confirmed by the slight shift in the imidazole bands and in the amide-associated C=O stretching.

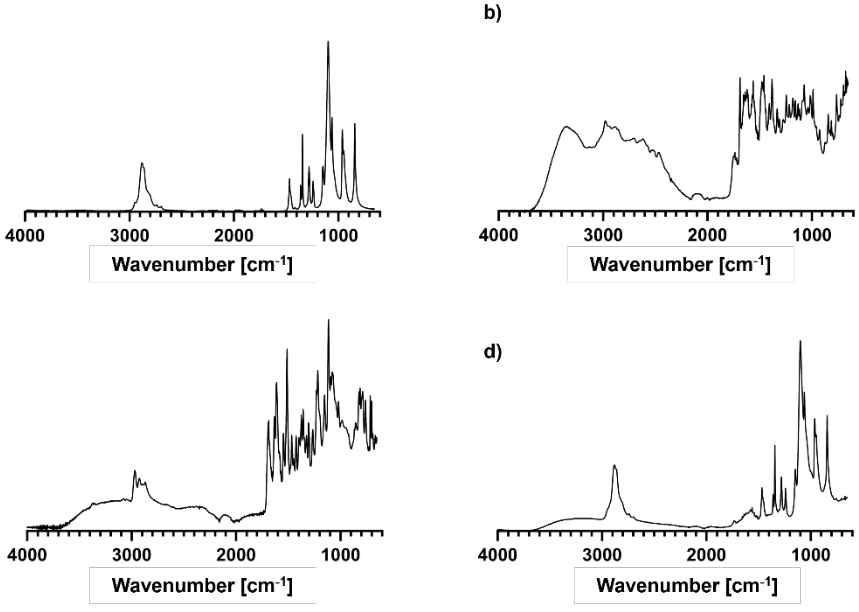


**Figure S3.** ATR-FTIR spectra of (a) COOH-PEG-COOH, (b) His-Rhod, (c) PAH-HA NG tagged with His-Rhod using $Co^{3+}$, (d) PAH-PEG NGs tagged with His-Rhod using $Co^{3+}$.

*His-Rhod $^1$H-NMR spectrum*

In Figure S4, the $^1$H-NMR spectrum of Rhod functionalized with histidine and used for His-tag conjugation of NGs is reported [1,2].

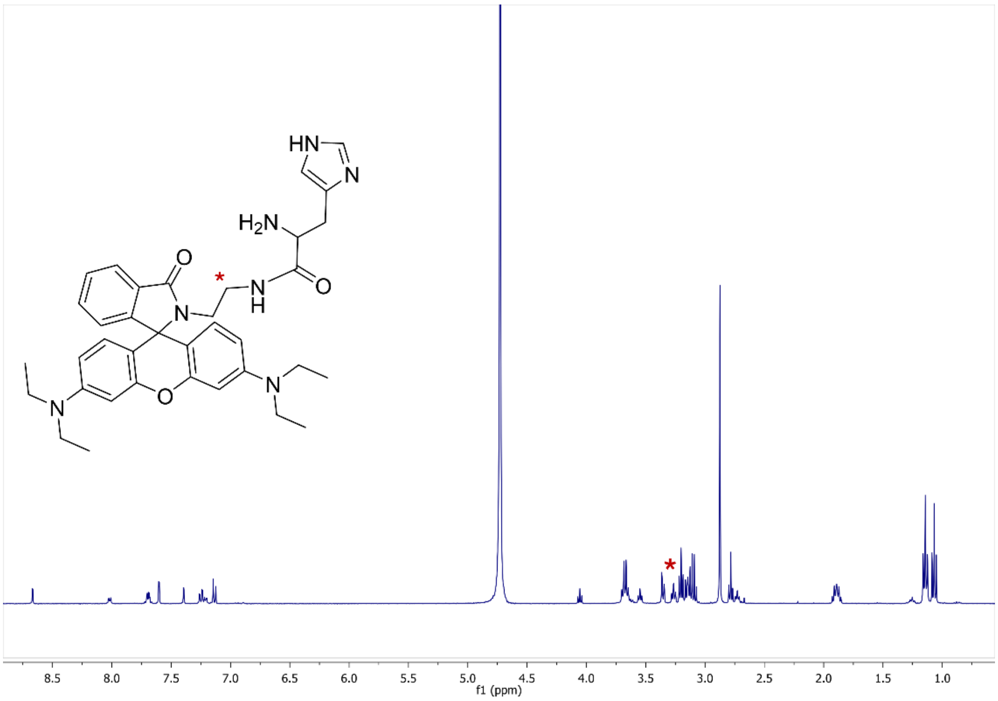


**Figure S4.** $^1$H-NMR spectrum of His-Rhod. Analysis was performed in $D_2O$. The shift of the -$CH_2$-NH- protons vicinal to the amide linkage is highlighted (*, in red).

*XRD analysis for metal complexation*

Figure S5 shows the XRD characterization of the NGs after metal complexation ($Ni^{2+}$ and $Co^{3+}$).

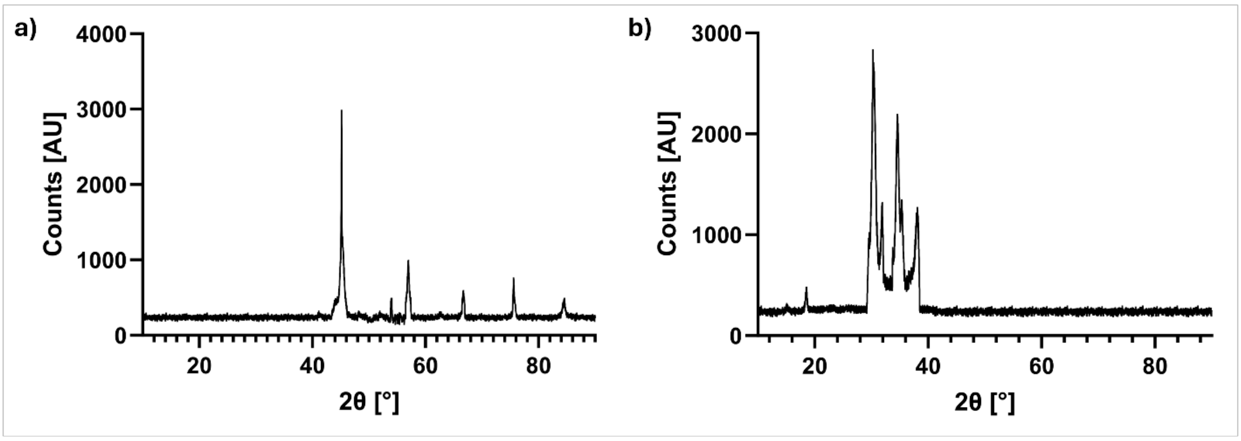


**Figure S5.** XRD characterization of NGs after metal complexation step: (a) after $Ni^{2+}$ complexation, (b) after $Co^{3+}$ complexation.

*Colloidal stability of His-Rhod conjugated system*

To investigate the influence of His-Rhod conjugation on the colloidal stability of the NGs, DLS analysis was performed over 48h, both in DDIW and PBS supplemented with 10% fetal bovine serum (FBS) as a physiologically relevant medium. As shown in Figure S6, no significant change in NG size was observed over time, within each medium, confirming the stability of the nanoarchitecture. Nevertheless, NG hydrodynamic diameter was found to be smaller in PBS-FBS, compared to DDIW. This effect could be related to the presence of salts, that interact with the ionizable groups present on the surface of the NGs [3], and protein-polymer interactions which could modulate the network conformation.
Regarding ζ-potential analysis, NGs showed no significant variations in charge over 48h (Figure S7).

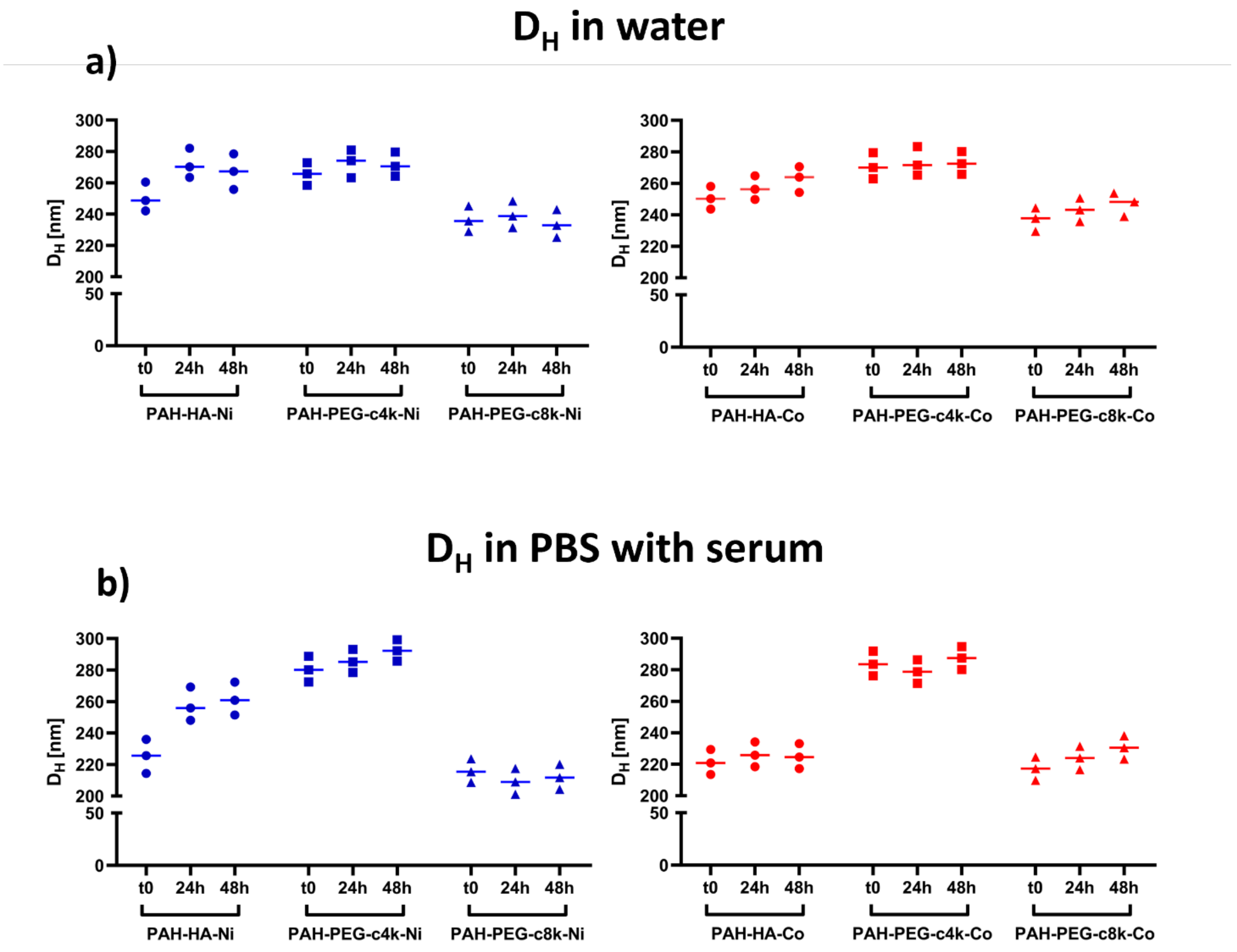


**Figure S6:** DLS results of His-Rhod conjugated NGs at $t_0$, 24h and 48h. Analysis was performed in triplicate. Results are reported in DDIW (a) and PBS with serum (b).

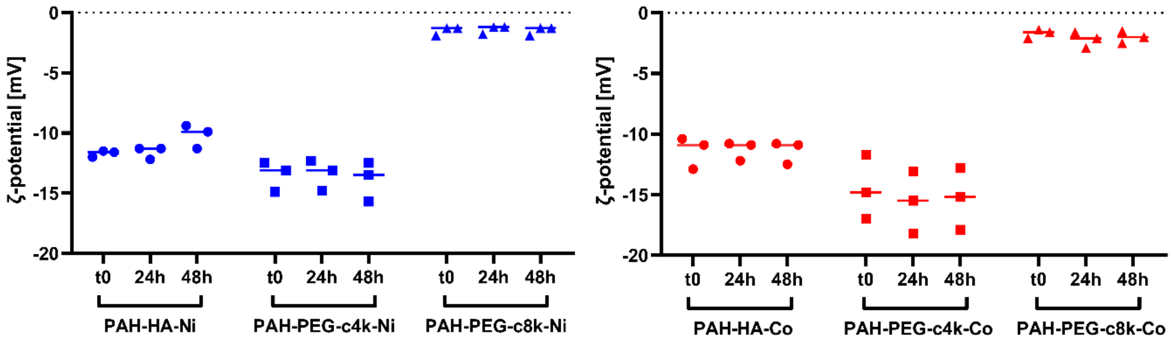


**Figure S7.** ζ-potential results of His-rhod conjugated NGs in DDIW at $t_0$, 24h and 48h. Analysis was performed in triplicate.

*TEM images of NGs*

Transmission electron microscopy (TEM) micrographs of NG PAH-HA, NG PAH-PEG-c4k and NG PAH-PEG-c8k, were acquired with Philips CM200 FEG (field emission gun) at 200 kV.
Sample was prepared by depositing a drop of the NG dispersion was deposited on a carbon-coated copper grid and air dried at room temperature.

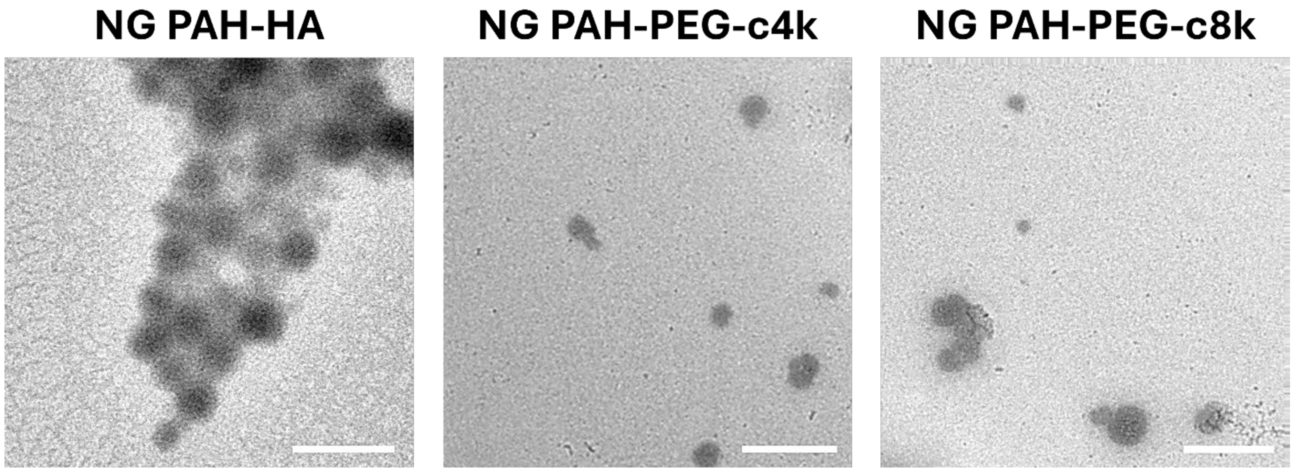


**Figure S8.** Representative TEM images of NGs, acquired at 200 kV. Scalebar = 300 nm.

*Flow cytometry of OVCAR3 with NGs complexed with $Ni^{2+}$*

Flow cytometry analysis performed on OVCAR3 cells following the administration of $Ni^{2+}$-complexed NGs, prepared with a lys-NTA:His-Rhod molar ratio equal to 1:1, is reported in Figure S9. The obtained results, in terms of NG internalization and apoptosis activation, are similar to those observed for the $Co^{3+}$-based formulations.

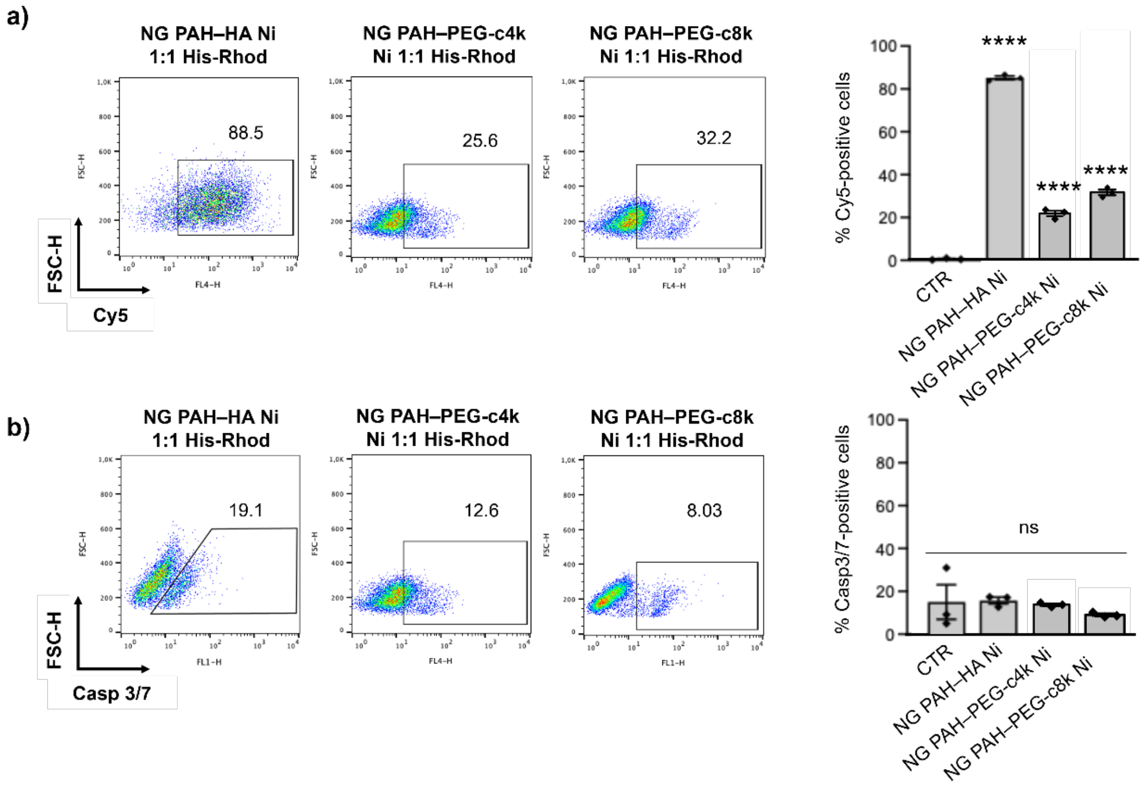


**Figure S9.** Uptake of NGs and apoptosis activation in OVCAR3 cells. Cells were incubated for 24 hours with medium (CTR) or the indicated Cy5-labelled NGs (0.1 mg $ml^{-1}$) after the Ni-mediated His-Rhod conjugation. At the end of incubation, NG engulfment (a) and caspase 3/7 (Casp3/7) activation (b) were analysed by flow cytometry. The percentages of Cy5-positive cells and Casp3/7-positive cells were calculated. Data shown mean ± SEM of triplicate cultures. Statistical significance was determined by Student's t test. (*) $p < 0.05$, (**) $p < 0.01$, (***) $p < 0.001$, (****) $p < 0.0001$, ns = not significant.

Figures S10 and S11 show the flow cytometry analysis related to the administration of His-Rhod NGs prepared using a lys-NTA:His-Rhod molar ratio equal to 1:10. For PEG-based specimens (Figure S10), the NG internalization is consistent with the samples prepared at lys-NTA:His-Rhod 1:1 as well as the absence of relevant cytotoxic effects. Conversely, the administration of HA-based NGs (Figure S11) results in an apoptosis effect.

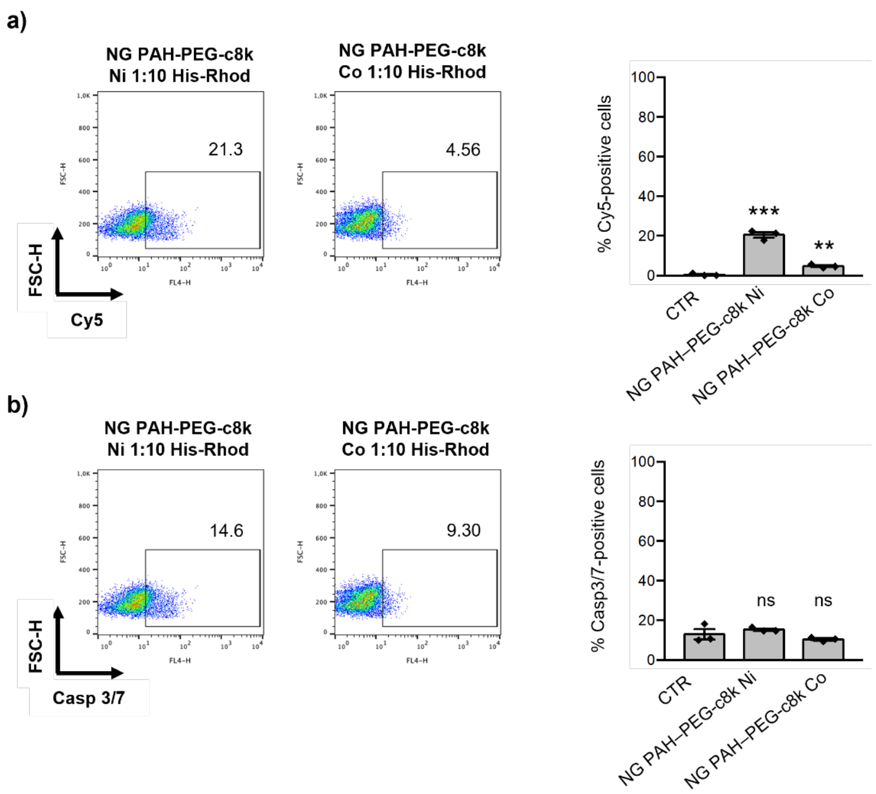


**Figure S10.** Uptake of NGs and apoptosis activation in OVCAR3 cells. Cells were incubated for 24 hours with medium (CTR) or the indicated Cy5-labelled NGs (0.1 mg $ml^{-1}$). At the end of incubation, NG engulfment (a) and Casp3/7 activation (b) were analyzed by flow cytometry. The percentages of Cy5-positive cells (a) and Casp3/7-positive cells (b) were calculated. Data shown mean ± SEM of triplicate cultures. Statistical significance was determined by Student's t test. (*) $p < 0.05$, (**) $p < 0.01$, (***) $p < 0.001$, (****) $p < 0.0001$, ns = not significant.

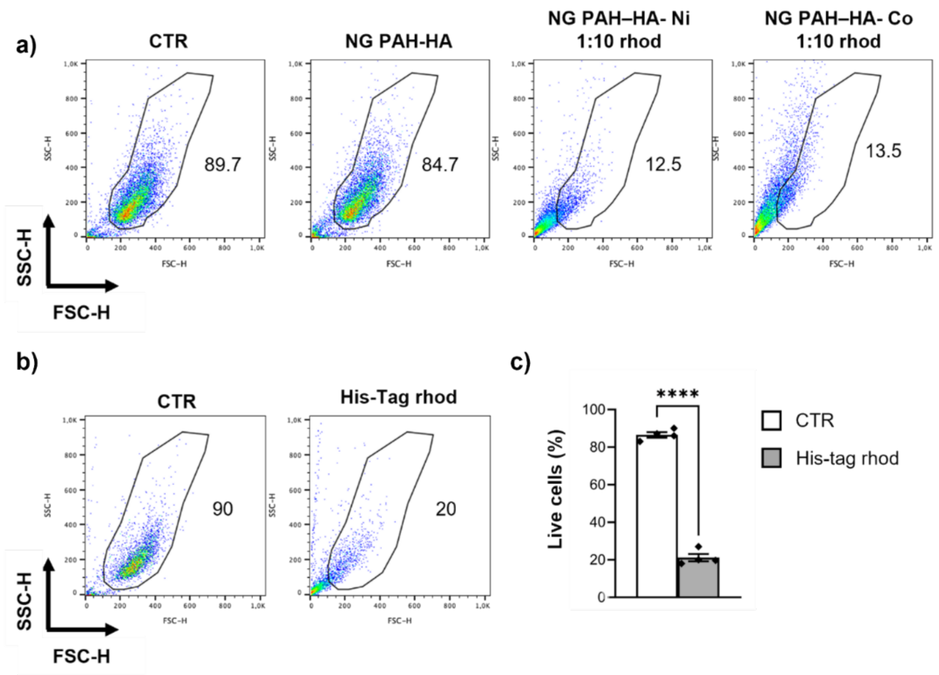


**Figure S11.** Flow cytometry analysis of apoptosis induction in OVCAR3 cells cultured for 24 hours with medium (CTR) or PAH-HA NGs prepared with a lys-NTA:His-Rhod molar ratio 1:10 (a), or free His-Tag rhod at the same concentration present in PAH-HA NGs (b,c) by measuring the reduction in cell size (FSC-H).

*6xHis complex verification*

To validate the effectiveness of the proposed procedure a recombinant bispecific antibody CD28xMUC1 (#BSFAB-H236, Creative Biolabs, Brookhaven, NY, USA), presenting 6xHis moieties in its structure, was used. Recombinant bispecific antibodies (BsABs) targeting CD28 co-stimulatory receptor on T lymphocytes and tumour-associated antigens (TAA) are emerging as a useful immunotherapeutic tool to elicit tumour-specific immunity [4]. As a proof-of-concept, we developed NGs conjugated to the recombinant BsAb targeting CD28 and mucin 1 (MUC1), a TAA highly expressed on ovarian cancer [5].
In particular, we tested the BsAb His-tagging via Co-mediated coordination in Cy5-labelled PAH-PEG-c8k NGs, following the same procedure presented in Section 2.10. A molar ratio His-sites : NTA 1:1 was used, based on the BsAb molecular weight specifications provided by the manufacturer.
To analyse the surface binding of NGs, OVCAR3 cells were incubated for 25 min at room temperature with culture medium (Ctr), control Cy5-NGs (0.02 mg/ml) or Cy5-NGs conjugated with CD28xMUC1 (Tandem Fab) BsAbs (#BSFAB-H236, Creative Biolabs, USA) (0.02 mg/ml).
After fixation with 3% paraformaldehyde, cells were seeded onto poly-L-lysine–coated coverslips, permeabilised, and analysed by confocal microscopy (Zeiss LSM 900). Nuclei were stained with DAPI and the NG detected exploiting the Cy5 fluorescence intensities. Surface-bound NGs were quantified using Fiji ImageJ software and expressed as relative intensity.
Figure S12a shows the confocal micrographs confirming the interaction between decorated NGs (in red) and cells. results obtained with a fluorescent microscope (Zeiss LSM 900, Carl Zeiss AG, Oberkochen, DE), highlighting the presence of the antibody grafted on the HA-PEG NG (in red), distributed on the OVCAR3 cells nuclei.
The analysis of the binding of NGs and CD28xMUC1-functionalized NGs on the surface of MUC1-positive OVCAR3 cells (Figure S12a) revealed a binding of control NGs, which was markedly increased after conjugation with CD28xMUC1 BsAbs, as reported in Figure S12b.

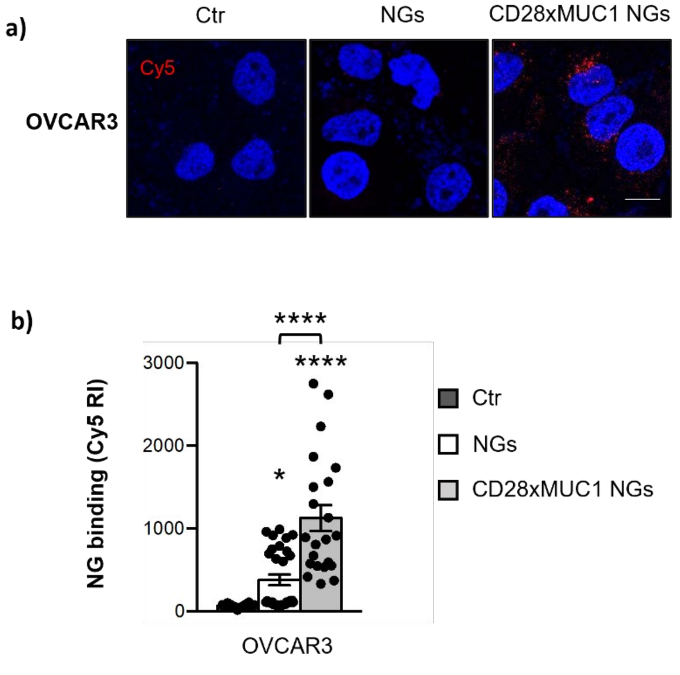


**Figure S12.** (a) Representative microscopy images of OVCAR3 cells incubated for 25 min with culture medium (Ctr), control Cy5-NGs (NGs, 20 μg $ml^{-1}$), or BsAb decorated Cy5-NGs (CD28xMUC1 NGs, 20 μg $ml^{-1}$). Nuclei were stained with DAPI. Scale bar = 20 μm. (b) NG binding: the Cy5 fluorescence intensities of surface-bound NGs were quantified and expressed as relative intensity (RI). Data show mean ± SEM of at least 24 cells. Statistical significance was calculated by one-way ANOVA. *$p < 0.05$, ****$p < 0.0001$.

This highlights how the methodology presented in this study can be applied effectively also to antibodies (or potentially other molecules) presenting a gold-standard 6xHis group in their structure, paving the way to a standardized method to functionalize polymeric NGs surface.

## References


(1) Shekari, Z.; Younesi, H.; Heydari, A.; Tajbakhsh, M.; Chaichi, M. J.; Shahbazi, A.; Saberi, D. Fluorescence Chemosensory Determination of Cu2+ Using a New Rhodamine–Morpholine Conjugate. *Chemosensors 2017, Vol. 5, Page 26* **2017**, *5* (3), 26. https://doi.org/10.3390/CHEMOSENSORS5030026.

(2) Zhang, Y. S.; Balamurugan, R.; Lin, J. C.; Fitriyani, S.; Liu, J. H.; Emelyanenko, A. Pd2+ Fluorescent Sensors Based on Amino and Imino Derivatives of Rhodamine and Improvement of Water Solubility by the Formation of Inclusion Complexes with β-Cyclodextrin. *Analyst* **2017**, *142* (9), 1536–1544. https://doi.org/10.1039/C6AN02594C.

(3) Yuan, C.; Jin, S.; Wei, J.; Huang, J.; Liu, C.; Lei, X.; Zuo, Y.; Li, J.; Li, Y. The Shrinking Behavior, Mechanism and Anti-Shrinkage Resolution of an Electrospun PLGA Membrane. *J. Mater. Chem. B* **2021**, *9* (29), 5861–5868. https://doi.org/10.1039/d1tb00734c.

(4) Lotze, M. T.; Olejniczak, S. H.; Skokos, D. CD28 Co-Stimulation: Novel Insights and Applications in Cancer Immunotherapy. *Nat. Rev. Immunol.* **2024**, *24* (12), 878–895. https://doi.org/10.1038/S41577-024-01061-1.

(5) Deng, J.; Wang, L.; Chen, H.; Li, L.; Ma, Y.; Ni, J.; Li, Y. The Role of Tumour-Associated MUC1 in Epithelial Ovarian Cancer Metastasis and Progression. *Cancer Metastasis Rev.* **2013**, *32* (3–4), 535–551. https://doi.org/10.1007/S10555-013-9423-Y.
